# Contrasting Strategies for Sucrose Utilization in a Floral Yeast Clade

**DOI:** 10.1128/msphere.00035-22

**Published:** 2022-03-31

**Authors:** Carla Gonçalves, Margarida Marques, Paula Gonçalves

**Affiliations:** a Associate Laboratory i4HB—Institute for Health and Bioeconomy, NOVA School of Science and Technology, Universidade NOVA de Lisboa, Caparica, Portugal; b UCIBIO—Applied Molecular Biosciences Unit, Department of Life Sciences, NOVA School of Science and Technology, Universidade NOVA de Lisboa, Caparica, Portugal; University of Georgia

**Keywords:** W/S clade, alpha-glucosidase, comparative genomics, gene cluster, horizontal gene transfer, invertase, sucrose utilization

## Abstract

Yeast species in the *Wickerhamiella* and *Starmerella* genera (W/S clade) thrive in the sugar-rich floral niche. We have previously shown that species belonging to this clade harbor an unparalleled number of genes of bacterial origin, among which is the *SUC2* gene, encoding a sucrose-hydrolyzing enzyme. In this study, we used complementary *in silico* and experimental approaches to examine sucrose utilization in a broader cohort of species representing extant diversity in the W/S clade. Distinct strategies and modes of sucrose assimilation were unveiled, involving either extracellular sucrose hydrolysis through secreted bacterial Suc2 or intracellular assimilation using broad-substrate-range α-glucoside/H^+^ symporters and α-glucosidases. The intracellular pathway is encoded in two types of gene clusters reminiscent of the *MAL* clusters in Saccharomyces cerevisiae, where they are involved in maltose utilization. The genes composing each of the two types of *MAL* clusters found in the W/S clade have disparate evolutionary histories, suggesting that they formed *de novo*. Both transporters and glucosidases were shown to be functional and additionally involved in the metabolization of other disaccharides, such as maltose and melezitose. In one *Wickerhamiella* species lacking the α-glucoside transporter, maltose assimilation is accomplished extracellularly, an attribute which has been rarely observed in fungi. Sucrose assimilation in *Wickerhamiella* generally escaped both glucose repression and the need for an activator and is thus essentially constitutive, which is consistent with the abundance of both glucose and sucrose in the floral niche. The notable plasticity associated with disaccharide utilization in the W/S clade is discussed in the context of ecological implications and energy metabolism.

**IMPORTANCE** Microbes usually have flexible metabolic capabilities and are able to use different compounds to meet their needs. The yeasts belonging to the *Wickerhamiella* and *Starmerella* genera (forming the so-called W/S clade) are usually found in flowers or insects that visit flowers and are known for having acquired many genes from bacteria by a process called horizontal gene transfer. One such gene, dubbed *SUC2*, is used to assimilate sucrose, which is one of the most abundant sugars in floral nectar. Here, we show that different lineages within the W/S clade used different solutions for sucrose utilization that dispensed *SUC2* and differed in their energy requirements, in their capacity to scavenge small amounts of sucrose from the environment, and in the potential for sharing this resource with other microbial species. We posit that this plasticity is possibly dictated by adaptation to the specific requirements of each species.

## INTRODUCTION

Microbes are exposed to frequent changes in their environment and must as a result be constantly able to adapt to those changes, which may imply fine-tuning of various cellular processes. Among these, adjustments of the metabolic toolkit to the nutritional sources available and responses to environmental stress, for example, occur mainly through modulation of gene expression. For metabolic genes, this often implies activation of the expression of genes involved in the utilization of available carbon sources and repression of genes contributing to metabolize alternative compounds. Like in many other microbes, in ascomycetous yeasts this may also involve a choice between different modes of energy conservation (respiration versus fermentation) (1–3) and also a modulation of the type of nutrient transporter operating under each condition, since transporters vary widely in affinity for their substrates and energy requirements (4).

Ascomycetous yeasts have some of the most compact genomes among eukaryotes, with short intergenic regions and few introns (5). Comparative genomics has shown over the past decade that each species tends to carry only a limited subset of the complete repertoire of metabolic tools identified in yeasts, presumably including only the genes/pathways with the most favorable impact on fitness in their particular niche. Hence, the metabolic genetic toolkit will tend to be more extended in ubiquitous species (i.e., not strongly associated with a particular niche) than in species circumscribed to a certain habitat. Notably, adaptation to nutrient-rich environments is likely to entail loss of genome content because of the dispensability of certain biosynthetic or catabolic functions. An extreme instance of this kind of genome reduction is observed in intracellula pathogens that tend to lack most metabolic genes (6, 7). This means that upon adaptation to a new environment, the attainment of optimal fitness is likely to entail both the shedding of dispensable genes/pathways and the acquisition of new metabolic capabilities, which means the acquisition of new genes or new gene functions.

In the model yeast Saccharomyces cerevisiae, genes that are not fixed in the species forming the so-called pan-genome are often located in subtelomeric regions, where gene content is highly variable. In certain cases, these genes are part of a cluster that constitutes the minimum requirement for a certain metabolic accomplishment (8). This is the case of the *MAL* gene clusters that contain genes encoding a maltase, a maltose transporter, and a regulator that participates in activation of the first two genes (9, 10). Other examples of subtelomeric genes in S. cerevisiae are those encoding two disaccharide extracellular hydrolases, namely, the ubiquitous Suc2 invertase catalyzing the cleavage of sucrose in glucose and fructose (11) and Mel1, which is responsible for the extracellular hydrolysis of melibiose and is found only in certain S. cerevisiae populations (12).

Several mechanisms may account for loss and acquisition of genes in yeasts. While sexual reproduction may suffice to account for redistribution within a species of genes that are not fixed, other mechanisms are required to explain the appearance of new metabolic competencies in any given microbial species when the required gene is absent from the gene pool of the species. In addition to *de novo* gene creation, less frequently observed, gene duplication followed by neofunctionalization (10, 13) and horizontal acquisition of genes (HGT) (3, 14–17) are mechanisms used to widen the scope of metabolic tools. Interestingly, gene loss was proposed to be adaptive in some cases (as opposed to being solely the result of genetic drift), possibly conferring a selective advantage by contributing to conserve limited cellular resources (18).

In recent years, the yeast clade formed by the *Wickerhamiella* and *Starmerella* genera (henceforth the W/S clade) stood out for the exorbitant number of horizontally acquired genes found in their genomes (3, 15, 17). These acquisitions concern numerous metabolic genes and were preceded by a massive loss of metabolic traits, both being possibly related to the adaptation of these yeasts to the floral niche by, for instance, allowing for vitamin (16) and iron (19) scavenging or maximizing the transport and metabolism of the highly abundant fructose (15).

In this study, we examined sucrose metabolism, an important trait in the W/S yeast clade because, along with glucose and fructose, this is one of the most abundant sugars in the floral niche where these yeasts are usually found. Our previous work uncovered the presence of a *SUC2* gene horizontally acquired from bacteria in several *Starmerella* species, and we confirmed that this gene was pivotal for sucrose assimilation in Starmerella bombicola (3). The present availability of additional genomes allowed us to assess the distribution of the *SUC2* gene in a wider range of W/S clade species, thereby showing that it is present almost exclusively in the *Starmerella* subclade. We used comparative genomics to show that most *Wickerhamiella* species capable of metabolizing sucrose can make use of either α-glucoside hydrolases of wide specificity and α-glucoside/H^+^ symporters, paired in gene clusters, or a highly unusual extracellular hydrolase. Disaccharide hydrolytic activity seems to generally escape glucose repression. Moreover, two gene clusters of different evolutionary origins were found in different *Wickerhamiella* species, and our evidence suggests that these clusters were formed *de novo.*

## RESULTS

### Sucrose utilization in the W/S clade and distribution of *SUC2*.

**FIG 1:**
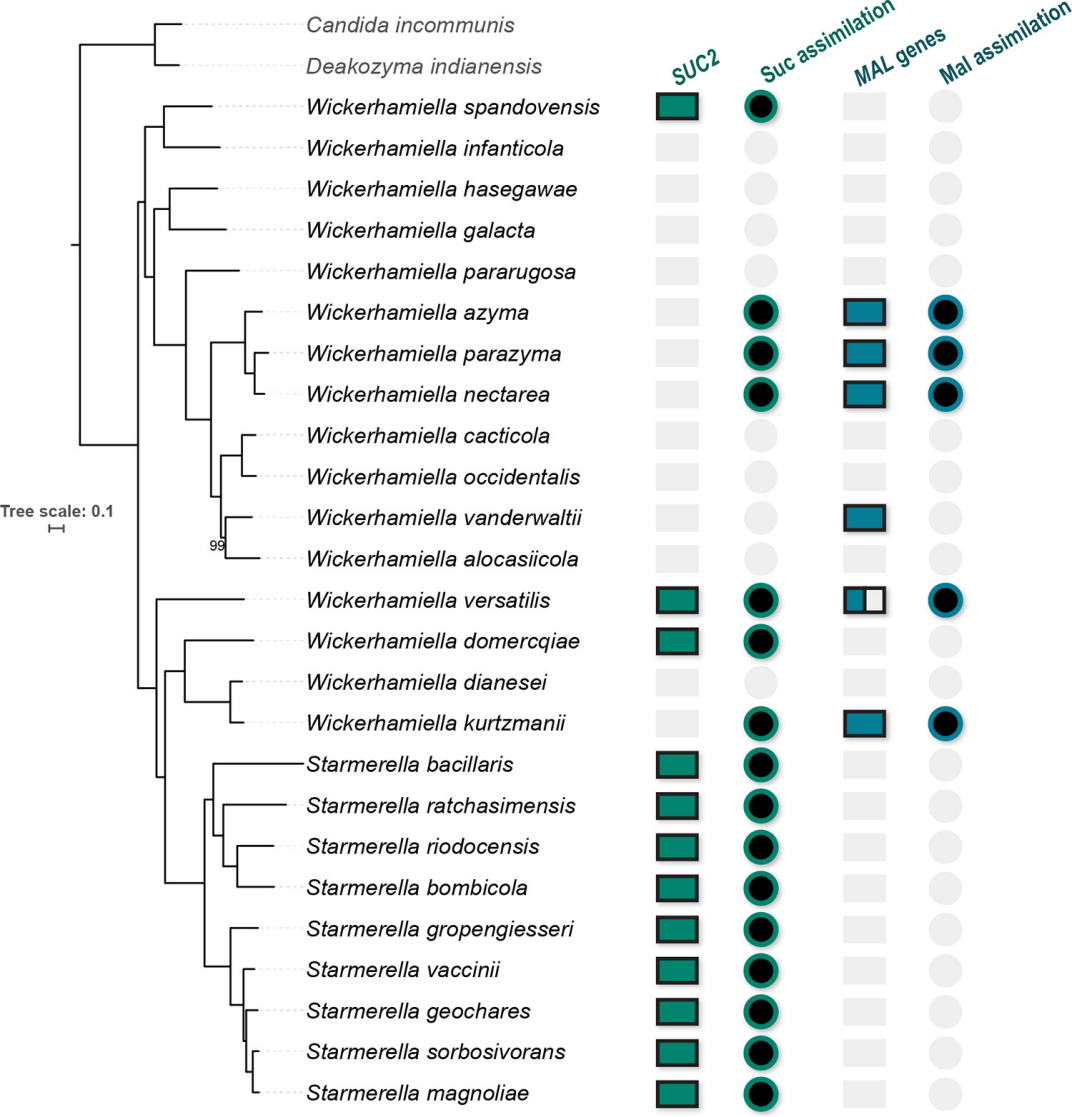
Distribution of sucrose utilization and sucrose utilization-related genes across 25 W/S clade species. The species phylogeny was constructed based on a concatenated alignment of 1,200 single-copy orthologs (SCO) obtained with Orthofinder 2, under the single LG+F+I+G4 model, using an ultrafast bootstrap strategy for branch support determination (only bootstrap values below 100 are indicated). Filled circles indicate detection of sucrose (green) or maltose (blue) utilization ability in the respective species. Filled squares indicate the presence of the SUC2 gene (green) or the *MAL* genes (*MAL*-*IMA* and *AGT*, in blue) in the respective genome. Gray squares/circles indicate that the respective feature was absent. For *Wickerhamiella versatilis*, the partially filled square represents an incomplete cluster, where only the gene encoding the α-glucosidase but not the transporter was found.

**FIG 2:**
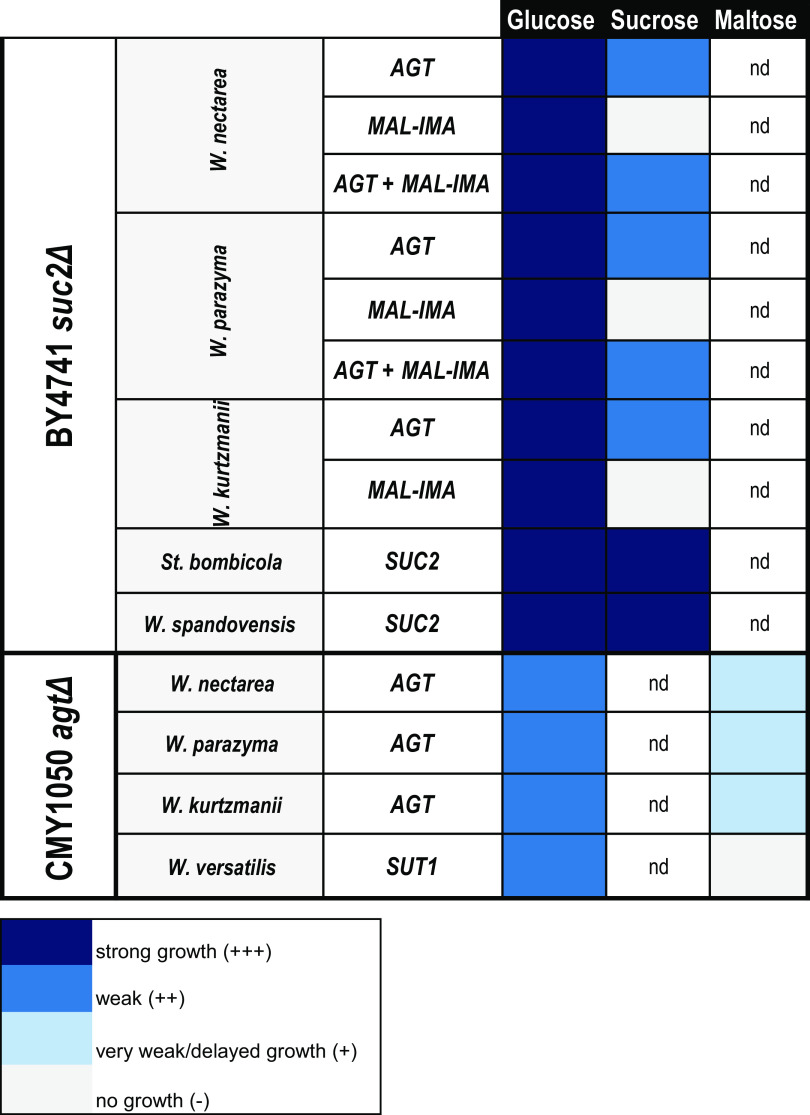
Functional analysis of W/S clade Mal-Ima and Agt homologs expressed in S. cerevisiae *suc*Δ and *agt*Δ strains. Growth was evaluated based on turbidity and on time required for the macroscopic observation of growth at 25°C: strong growth indicates that high turbidity was observed after 2 to 3 days of incubation, weak growth indicates that low turbidity was observed after 2 to 3 days of incubation, and delayed growth means that turbidity was observed only after more than 3 days of incubation. nd, not determined.

We previously described an invertase-encoding gene, *SUC2*, of bacterial origin in a few W/S clade species (3). This observation, and the fact that sucrose is an especially relevant substrate in the floral niche where these yeasts are usually found, led us to investigate how widespread sucrose utilization was in this clade, by scoring the ability of 25 *Starmerella* and *Wickerhamiella* species to grow on this carbon and energy source and inspecting for the presence of the *SUC2* gene in the respective genomes. All *Starmerella* species tested could grow on sucrose, but only 7 of the 16 *Wickerhamiella* species tested could use this substrate, in agreement with the literature (20, 21). As shown in Fig. 1 and in Data Set S1 in the supplemental material, there is an overall correlation between sucrose utilization and the presence of *SUC2*; however, four *Wickerhamiella* species lacking this gene were nevertheless able to metabolize sucrose. To confirm the function of the *SUC2*-encoded enzyme across the W/S clade phylogenetic range, we demonstrated the ability of both *St. bombicola* and Wickerhamiella spandovensis *SUC2* genes to complement an S. cerevisiae *suc2*Δ mutant (Fig. 2). In S. cerevisiae, the invertase encoded by the *SUC2* gene is an extracellular enzyme (11) and so are some of the bacterial invertases (22). *In silico* predictions complemented by experimental evidence indicated that all W/S clade invertases identified in this work are extracellular (Data Set S1). The distribution of *SUC2* and the phylogenetic position of the Suc2 protein from *W. spandovensis* (Fig. S1), which does not reflect the species phylogeny as usually observed for other HGT-derived genes in the W/S clade (3, 16, 19), suggests that the gene was acquired from bacteria by the ancestor of the *Starmerella* subclade and subsequently acquired by *W. spandovensis* from a *Starmerella*-related species.

10.1128/msphere.00035-22.1 DATA SET S1 Summary of growth test results on various α-glucosides for all W/S clade species studied in this work, presence and absence of genes related to sucrose utilization in the respective genomes, and *in silico* (SignalP and DeepLoc) and *in vivo* analyses of the cellular localization of Suc2. Ability to grow on sucrose, maltose, melezitose, and palatinose was tested as described in Materials and Methods in the main text. To determine the cellular localization of Suc2 invertases in W/S clade species, *in silico* prediction was obtained using SignalP v.5.0, which predicts the presence of signal peptide signature sequences, and DeepLoc, which predicts the protein subcellular localization. As some Suc2 proteins were predicted to be located in the intracellular space (cytoplasm, lysosome, or mitochondria), suggesting that sucrose metabolization would occur intracellularly, the supernatant cultures of the respective species were incubated with ∼50 g/L of sucrose and appearance of glucose and fructose was determined through HPLC analysis. The results are shown in the last column. Download Data Set S1, XLSX file, 0.8 MB. Copyright © 2022 Gonçalves et al. 2022 Gonçalves et al. https://creativecommons.org/licenses/by/4.0/ This content is distributed under the terms of the Creative Commons Attribution 4.0 International license.

10.1128/msphere.00035-22.4 FIG S1 Complete (left) and pruned (right) maximum likelihood phylogeny of Suc2 proteins. Branches are colored according to taxonomy as described in the key. On the pruned tree, fungal branches were collapsed for readability (270 sequences). Original sequences, alignment and tree files can be found in Figshare (https://figshare.com/articles/dataset/Phylogeny_Files/17695643). Download FIG S1, JPG file, 0.6 MB. Copyright © 2022 Gonçalves et al. 2022 Gonçalves et al. https://creativecommons.org/licenses/by/4.0/ This content is distributed under the terms of the Creative Commons Attribution 4.0 International license.

### *SUC2*-independent sucrose utilization in *Wickerhamiella*.

**FIG 3:**
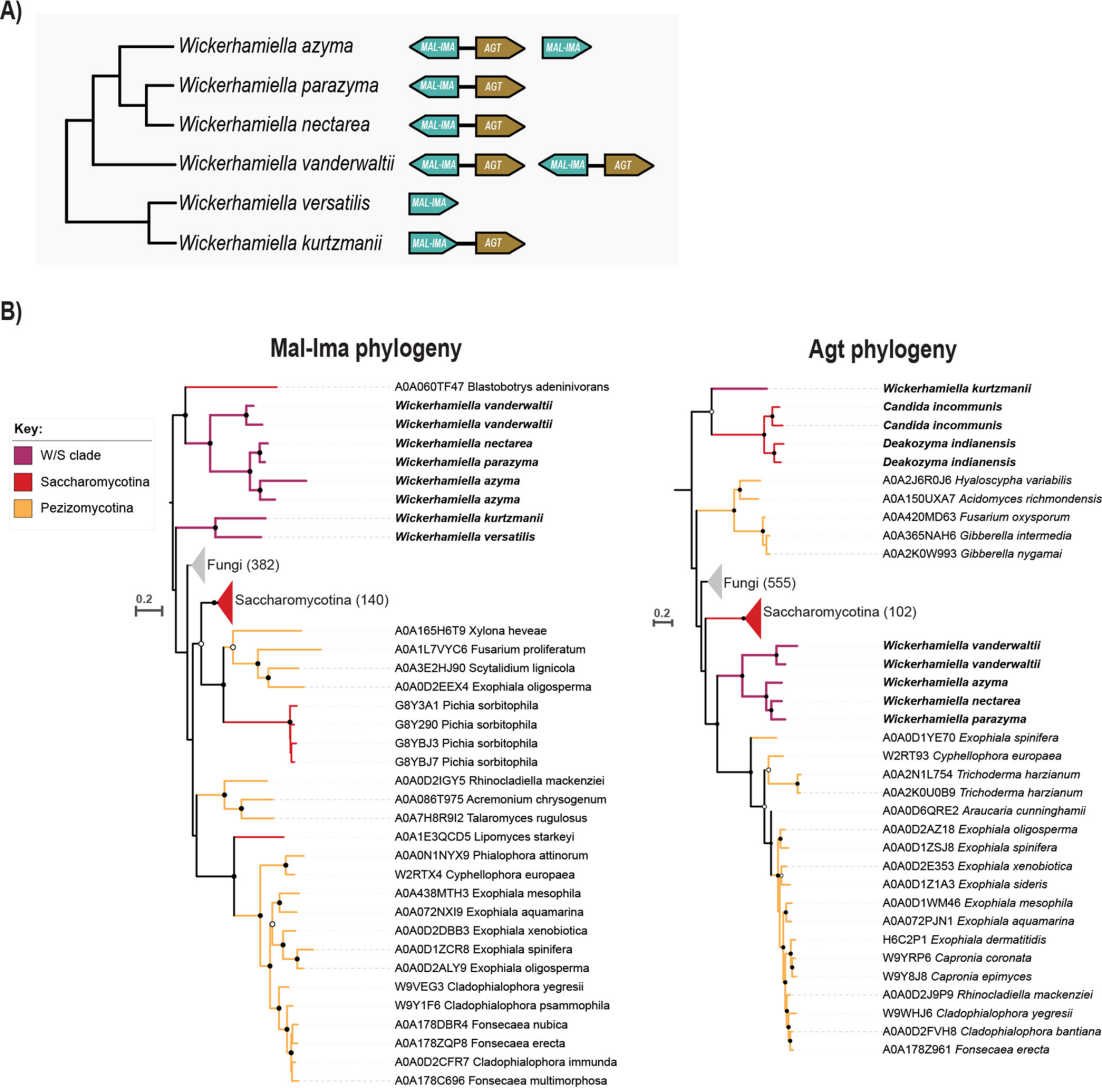
Gene organization and phylogenetic analyses of *MAL* genes. (A) Organization of *MAL* clusters in W/S clade species indicating direction of transcription for each gene. A detailed synteny analysis is shown in Fig. S3. (B) Pruned phylogenies of Mal-Ima α-glucosidases and Agt-like transporters. Branch support (ultrafast bootstrap) is indicated with dots: >95%, gray filled dots, and >99%, black dots. Reference Ima and Agt proteins from S. cerevisiae are clustered within the Saccharomycotina collapsed branch (in red). Branches were collapsed for readability, and the number of sequences in each collapsed branch is indicated in brackets. Complete phylogeny and alignment files can be found in Figshare (https://figshare.com/articles/dataset/Phylogeny_Files/17695643).

Given the distribution of the *SUC2* gene, the ability of four *Wickerhamiella* species lacking *SUC2* to use sucrose should be explained by the presence of alternative genes enabling sucrose metabolism. The other sucrose-hydrolyzing enzymes reported in yeasts are the α-glucosidases mainly associated with maltose metabolism in S. cerevisiae (11, 23, 24). Therefore, we surveyed the genomes of these species for genes encoding yeast α-glucosidase and α-glucoside transporters. The findings, summarized in Fig. 1 and Data Set S2, suggest that the four sucrose-utilizing *Wickerhamiella* species lacking SUC2, harbor instead α-glucosidase genes that closely resembles maltase genes found in filamentous fungi. In three species, W. azyma, W. nectarea, and W. parazyma, the gene is located next to a putative α-glucoside transporter, with both genes possibly sharing the same promoter in an arrangement reminiscent of the *MAL* gene clusters found in S. cerevisiae but lacking an activator gene (Fig. 3A).

10.1128/msphere.00035-22.2 DATA SET S2 Putative α-glucosidase and α-glucoside transporters found in W/S clade genomes. A local BLAST database was constructed with the predicted proteomes of W/S clade species and outgroups *Candida incommunis* and *Blastobotrys adeninivorans*. Putative α-glucosidase and α-glucoside/sucrose transporters in yeasts were obtained from UniProtKB (searches: “alpha glucosidase yeast,” “alpha glucoside transporter yeast,” and “sucrose transporter yeast”). Two local BLASTP searches were performed using all putative α-glucosidase protein sequences (total of 1,113 sequences) and all putative sucrose/α-glucoside transporters (total of 139 sequences) against the proteome database using an E value cutoff of 1e^−3^. The resulting proteins are shown below with the respective BLAST results against NCBI database. Putative maltases and α-glucosidases are highlighted in yellow, invertase is highlighted in blue, and a putative new sucrose transporter (Sut1-like) is highlighted in gray-blue. Download Data Set S2, XLSX file, 0.07 MB. Copyright © 2022 Gonçalves et al. 2022 Gonçalves et al. https://creativecommons.org/licenses/by/4.0/ This content is distributed under the terms of the Creative Commons Attribution 4.0 International license.

10.1128/msphere.00035-22.6 FIG S3 Gene content and organization in the *MAL*-*IMA*/*AGT* locus in W/S clade species (A) and the closest relatives, *Candida incommunis* and *Deakozyma indianensis* (B). Chromosomal regions encompassing *MAL* genes are indicated by gray bars for the species represented on the left. *MAL*-related genes are depicted as purple arrows in all species, indicating transcriptional orientation. Syntenic genes are represented in red (*W. nectarea* and *W. parazyma*), while nonsyntenic genes are colored in white, genes of bacterial origin are colored in blue (*W. versatilis*), and genes with unknown function are represented in gray. Download FIG S3, JPG file, 0.2 MB. Copyright © 2022 Gonçalves et al. 2022 Gonçalves et al. https://creativecommons.org/licenses/by/4.0/ This content is distributed under the terms of the Creative Commons Attribution 4.0 International license.

The cluster is apparently absent in the genomes of species that possess *SUC2* and of species that failed to grow on sucrose. It was found in W. kurtzmanii, which can grow on sucrose, and in W. vanderwaltii, which harbors two clusters but is nevertheless unable to grow on sucrose (Fig. 1; Data Set S2). In what concerns the predicted amino acid sequence at the active site, the W/S clade α-glucosidases are too divergent from others already characterized to warrant firm conclusions, but exhibit a threonine residue conserved in maltases but not in isomaltases (9, 10, 25) (Fig. S2).

10.1128/msphere.00035-22.5 FIG S2 Amino acid signature of W/S clade α-glucosidases highlighting the conservation of the threonine residue in the position 216 (according to S. cerevisiae Ima1). Amino acid signatures of other yeast α-glucosidases are also shown, including the *B. adeninivorans* Ag2 protein, which was shown to be the closest relative to Mal-Ima proteins from *W. azyma*, *W. parazyma*, and *W. nectarea* (Fig. 3 in the main text). The activity (maltase/isomaltase) predicted from the amino acid signature for each protein is also shown. Download FIG S2, JPG file, 0.1 MB. Copyright © 2022 Gonçalves et al. 2022 Gonçalves et al. https://creativecommons.org/licenses/by/4.0/ This content is distributed under the terms of the Creative Commons Attribution 4.0 International license.

The putative α-glucosidases identified in W/S clade species would be expected to accept also other sugar substrates, most notably maltose. For that reason, we evaluated maltose utilization in the W/S clade species included in this study and found that it was circumscribed to the α-glucosidase-harboring species (Fig. 1), again with the exception of *W. vanderwaltii*. This observation is consistent with maltose and sucrose being metabolized by the same enzyme in these species. Notably, in the Wickerhamiella versatilis genome, we were only able to find one α-glucosidase gene and not a flanking transporter, leaving open the question of how maltose transport is accomplished in this species. We also evaluated the ability of α-glucosidase-harboring species to metabolize other α-glucosides (melezitose and palatinose). Palatinose (isomaltose-like) and melezitose (maltose-like) were efficiently used by *W. nectarea*, *W. parazyma*, and *W. azyma* but not by *W. kurtzmanii* (Data Set S1), suggesting that most W/S clade enzymes are of the Mal-Ima type which accepts the entire disaccharide substrate range as the substrate (25).

Yeast α-glucosidases form a numerous family with complex patterns of evolution and many species in the Saccharomycotina harbor genes encoding multiple versions of the enzyme, while others have none. A phylogeny including the α-glucosidases most similar to W/S clade proteins, shows that sequences belonging to species of the *Wickerhamiella* subclade seem to be quite distinct from other yeast α-glucosidases, with the exception of a maltase encoded in the Blastobotrys adeninivorans genome, which is found in the same clade (Fig. 3B) and was shown to accept only maltose and related substrates (26).

Despite numerous attempts, it was not possible to obtain a phylogeny that could better address the question of the origin of the W/S clade Mal-Ima enzymes. Their proximity to the *Blastobotrys* enzyme leads us to postulate that it may be an ancient gene variant lost in other fungal lineages, most notably in other yeasts. Finally, the *W. kurtzmanii* and *W. versatilis* Mal-Ima proteins cluster together but apart from all other yeasts (Fig. 3B).

If the W/S clade α-glucosidase/transporter cluster was inherited as such, the phylogenetic positions of the Agt- and Mal-Ima-encoding genes are expected to be similar. Surprisingly, the Agt phylogenetic tree shows that the W/S clade proteins are found in two clades phylogenetically distant from each other (Fig. 3B) and clearly distinct from the phylogenetic positions of the respective Mal-Ima pair. The Agt transporters of *W. nectarea*, *W. azyma*, and *W. parazyma* cluster together in a clade that also includes *W. vanderwaltii* but no other yeasts, their closest relatives being transporters from the Pezizomycotina. On the other hand, the *W. kurtzmanii* Agt clusters with transporters belonging to two species which are the closest known relatives of the W/S clade (17), Candida incommunis and Deakozyma indianensis. However, the Mal-Ima protein from *W. kurtzmanii* does not cluster with any of the Mal-Ima proteins found in these two species. Moreover, in the *W. kurtzmanii* cluster, the two genes are transcribed in the same direction (Fig. 3A; Fig. S3), contrary to the organization of the clusters in the other W/S clade species examined.

### Activity and regulation of the Mal-Ima and Agt proteins.

To gain insight in the function of Mal-Ima and Agt proteins, the respective encoding genes were expressed in a *suc2*Δ S. cerevisiae strain. The results, summarized in Fig. 2, show that S. cerevisiae strains harboring only W/S clade Mal-Ima proteins were unable to grow on sucrose, suggesting that endogenous S. cerevisiae Agt transporters are not capable of efficient sucrose transport under the growth conditions used. On the contrary, heterologous expression of the Agt transporters alone supports weak growth on sucrose, indicating that they are functional in S. cerevisiae and that the endogenous S. cerevisiae Mal-Ima enzymes can ensure sufficient hydrolysis of sucrose once it is internalized. The simultaneous presence of plasmids encoding the cognate Mal-Ima enzymes with the plasmids encoding Agt transporters did not alter the weak growth phenotype on sucrose conferred by the transporters alone. Agt proteins were also expressed in an *agt*Δ S. cerevisiae strain (23) (Fig. 2), and all were able to support growth on maltose.

**FIG 4:**
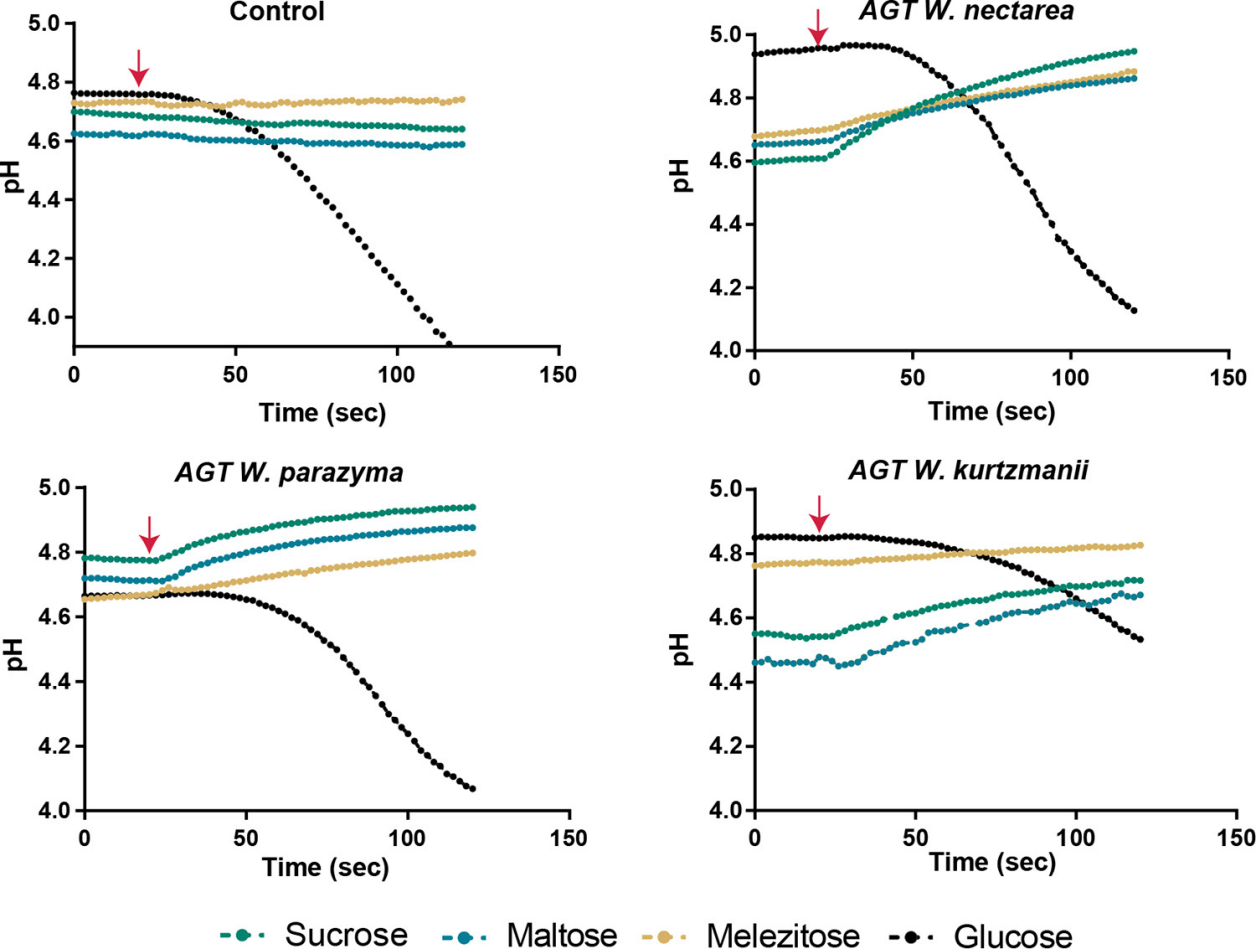
Activity of the Agt transporters from three *Wickerhamiella* species when heterologously expressed in the Saccharomyces cerevisiae *suc2*Δ strain. Symport activity was assessed by the measurement of the alkalinization of an unbuffered cell suspension of S. cerevisiae cells harboring p415GPD plasmids with each of the Agt-encoding genes from *W. nectarea*, *W. kurtzmanii*, and *W. parazyma*, elicited by the addition (marked by red arrows) of sucrose, maltose, or melezitose. Addition of each of the sugars tested was performed in turn. The first plot (control) represents a S. cerevisiae strain harboring the empty p415GPD plasmid (with no transporter gene). Glucose (black lines) was tested as an internal control of the viability of cell transport.

The *AGT1* gene encodes a general α-glucoside-H^+^ symporter in S. cerevisiae, meaning that α-glucoside transport is coupled to H^+^ uptake. We can therefore infer the specificity of the new Agt transporters by evaluating the alkalinization of an unbuffered cell suspension elicited by the addition of different α-glucosides (23, 27). In line with the observation that *W. parazyma* and *W. nectarea* can grow vigorously on sucrose-, maltose-, and melezitose-based media (Data Set S1), we could detect symport activity for the three different α-glucosides in S. cerevisiae recombinant strains expressing the Agt proteins from these two species (Fig. 4).

As for the S. cerevisiae strain expressing the Agt protein from *W. kurtzmanii*, melezitose failed to elicit a symport signal, but symport activity, albeit weak, was observed when sucrose and maltose were added to the cell suspension.

**FIG 5:**
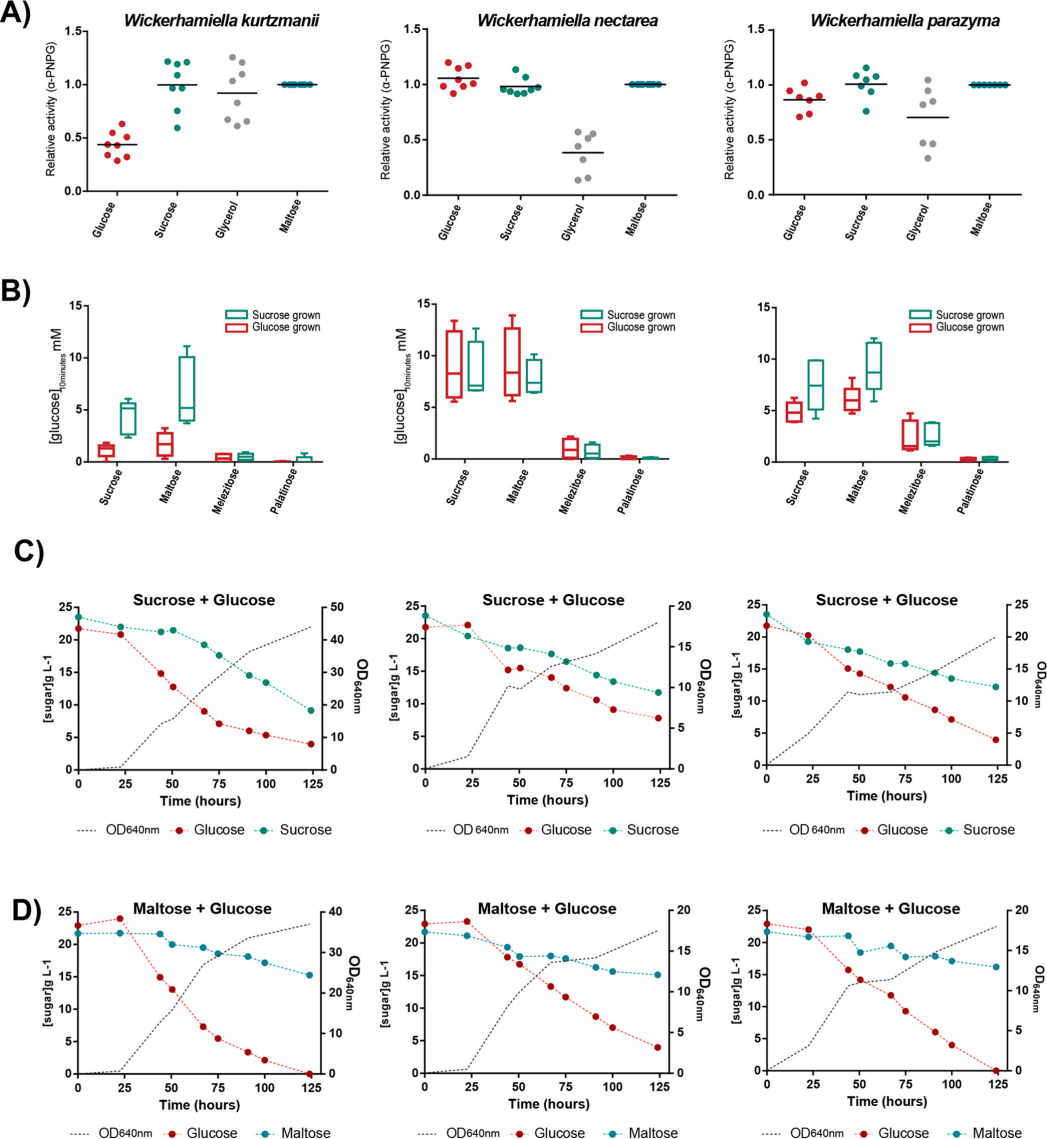
W/S clade α-glucosidase activities and sugar consumption profiles. (A) Relative α-glucosidase activities of cell extracts originated from cultures pregrown on glucose-, sucrose-, and glycerol-based medium compared to the α-glucosidase activity of cell extracts originated from cultures pregrown on maltose-based medium. At least three independent assays (two replicates each) are represented. (B) Concentration of glucose after 10 min of incubation of cell extracts of W/S clade species pregrown in glucose (red) or sucrose (green) with 250 mM concentrations of different glucosides (sucrose, maltose, melezitose, and palatinose). Minimum, maximum, and median values of, at least, three independent assays are represented. (C and D) Sugar consumption profiles of W/S clade species pregrown in a mixture of 2% (wt/vol) glucose and 2% (wt/vol) sucrose (C) or 2% (wt/vol) glucose and 2% (wt/vol) maltose (D). Assays were performed in triplicate, but only a representative assay is shown. Detection of α-glucosidase activity was inspected by collecting 2-mL samples of each growing culture at several time points (22 h, 30 h, 50 h, and 75 h).

We noted that unlike the S. cerevisiae *MAL* clusters and others found in various yeasts, none of the W/S clade *MAL* clusters included an activator, while in the closely related species *C. incommunis* and *D. indianensis*, a gene bearing some resemblance to the transcriptional activator *MAL13* was found next to maltase-encoding genes (Fig. 3A, Fig. S3, and Data Set S2). The absence of an activator raised the question of how expression of the cluster genes is regulated with respect to the available sugars. To address this question, we prepared cell extracts of *W. kurtzmanii*, *W. nectarea*, and *W. parazyma*, which have only one *MAL-IMA* gene, and assayed α-glucosidase activity, using a chromogenic substrate (4-Nitrophenyl α-D-glucopyranoside). In *W. parazyma*, similar enzymatic activities were measured when cells were pregrown on putative repressing (2% glucose) or nonrepressing (2% glycerol, 2% sucrose, and 2% maltose) carbon sources (Fig. 5A), leading to the conclusion that expression is subject neither to glucose repression nor to activation by a substrate. In *W. nectarea*, a similar pattern was observed, although the activity was lower when cells were pregrown on glycerol than under the remaining conditions (including glucose). This constitutive pattern of expression is consistent with the dispensability of the activator protein; however, in *W. kurtzmanii*, some evidence for glucose repression was found. We subsequently detected α-glucosidase activities in these species pregrown on sucrose and glucose using sucrose, maltose, melezitose, and palatinose as substrates. The results shown in Fig. 5B are consistent with sucrose and maltose being hydrolyzed by the same enzyme in all species and being the main substrates of these enzymes, while the activities using melezitose and palatinose as substrates were low or undetectable. We further confirmed the absence of glucose repression by growing the cells in mixtures of glucose and sucrose or glucose and maltose (Fig. 5C and D). Coconsumption of glucose and sucrose was observed in all species, confirming constitutive expression of the *MAL* genes, however maltose consumption was very slow, especially in *W. kurtzmanii* and *W. parazyma*. To confirm that under coconsumption conditions the genes encoding the ɑ-glucosidase were being expressed, the presence of α-glucosidase activity in cell extracts was measured at several time points (Fig. 4C and D) using α-PNPG, maltose, and sucrose as substrates. We detected α-glucosidase at all time points using the three substrates, which indicates that even in the apparent absence of maltose consumption, α-glucosidase is present, suggesting that impairment of maltose consumption might be related to inhibition of transport by glucose (28). Despite being able to grow on maltose, no α-glucosidase activity was detected in *W. versatilis* cell extracts when cells were pregrown under any of the conditions tested. Similarly, no activity could be measured using cell extracts of *W. vanderwaltii* pregrown in either glucose or glycerol, in agreement with its inability to grow in any of the α-glucosides tested (Fig. 1).

### *Wickerhamiella versatilis* harbors an extracellular maltase.

The phenotype of *W. versatilis* is intriguing because this species can grow on maltose while it apparently lacks a transporter. This could imply either that its Mal-Ima protein is secreted to the extracellular medium or that other α-glucoside transporters unrelated to Agt are present. In fact, among the putative α-glucoside transporters flagged in W/S clade genomes (Data Set S2), we could find a putative transporter bearing some resemblance to the sucrose/maltose symporter Sut1, from Schizosaccharomyces pombe (fission yeast) (29). However, the *SUT1*-like gene from *W. versatilis* failed to complement growth of the S. cerevisiae *agt*Δ strain on maltose and therefore might not explain the growth of *W. versatilis* on maltose (Fig. 2).

**FIG 6:**
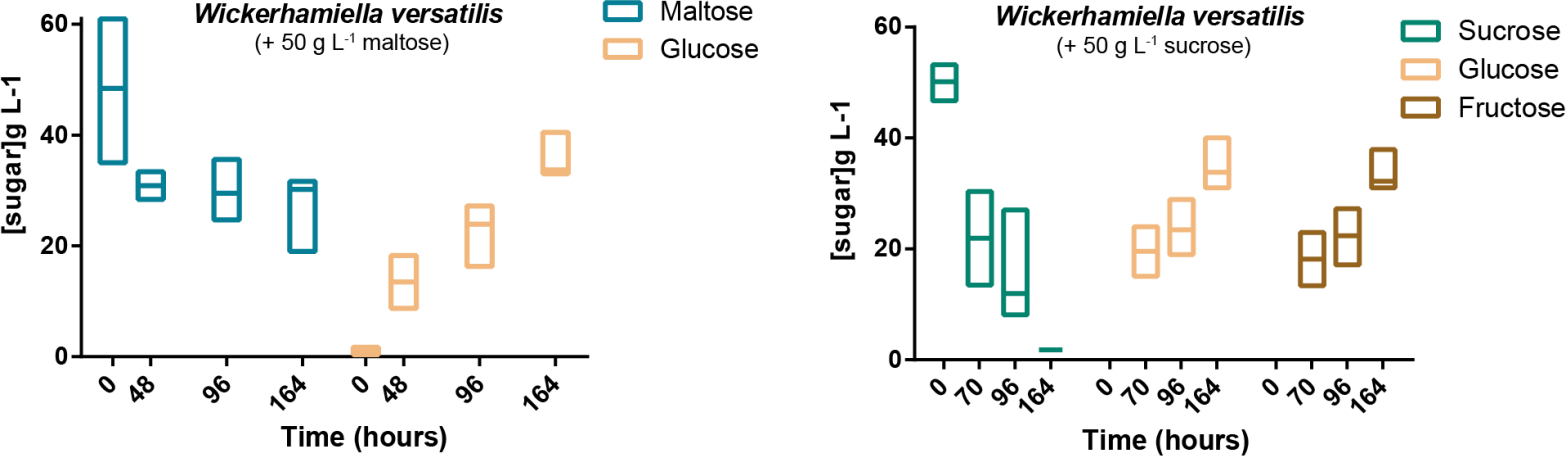
Detection of extracellular ɑ-glucosidase (left) and invertase (right) activities in culture supernatants of *Wickerhamiella versatilis*. Filtered culture supernatants were incubated with ∼50 g L^−1^ of sucrose (for invertase detection) or maltose (for α-glucosidase detection) for up to 164 h at 27°C with orbital shaking, and sugar concentration was determined by HPLC. At least three independent assays are represented (maximum, minimum, and median values are shown).

Even though *in silico* predictions failed to find evidence of extracellular localization of any of the Mal-Ima proteins (Data Set S1), we subsequently tested whether we could find evidence for extracellular α-glucosidase activity in cell-free supernatants of *W. versatilis* cultures pregrown in maltose-based medium. After incubation of the cell-free supernatants during up to 164 h with ∼50 g/L of maltose, glucose was detected and its concentration increased with incubation time (Fig. 6, left), while the cell extracts prepared from the same cultures lacked any α-glucosidase activity (Fig. S4). Sucrose hydrolysis observed for cell-free supernatants was foreseen (Fig. 6, right), because even in the absence of another extracellular invertase, these cells would be expected to express Suc2. For the phylogenetically related *W. kurtzmanii* and also for *W. nectarea*, no evidence for extracellular maltose hydrolysis was found in culture supernatants, while in cell extracts prepared from the respective cultures, ɑ-glucosidase activity could be readily detected (Fig. S4). Taken together, these results suggest that at least a significant fraction of the α-glucosidase from *W. versatilis* is secreted, which seems to be a unique feature among the W/S clade α-glucosidases.

10.1128/msphere.00035-22.7 FIG S4 Intracellular (A) and extracellular (B) detection of α-glucosidase activity in *W. versatilis*, *W*. *nectarea*, and *W*. *kurtzmanii*. (A) α-Glucosidase was assayed as described in Materials and Methods in the main text with α-PNPG, and the photo was taken 10 min (*W. nectarea* and *W. kurtzmanii*) or 20 min (*W. versatilis*) after the addition of α-PNPG. The yellow color indicates the presence of *p*-nitrophenol (originating from the breakdown of α-PNPG by the α-glucosidase). Two independent assays (A and B) are represented. At least three independent assays were performed for each species, but only two representative assays are shown for *W. nectarea* and *W. kurtzmanii* because different time points were sampled. The slight increase of maltose and sucrose concentration in *W. nectarea* and *W. kurtzmanii* (B) over time can be attributed to some degree of evaporation of the culture media during incubation at 27°C. Download FIG S4, JPG file, 0.3 MB. Copyright © 2022 Gonçalves et al. 2022 Gonçalves et al. https://creativecommons.org/licenses/by/4.0/ This content is distributed under the terms of the Creative Commons Attribution 4.0 International license.

## DISCUSSION

Yeasts included in the *Wickerhamiella* and *Starmerella* genera form a clade unique among the Saccharomycotina, harboring unusually high numbers of genes horizontally acquired from bacteria. Also, according to estimates concerning the evolution of metabolic traits in yeasts (Saccharomycotina), the ancestor of the W/S clade underwent a massive loss of metabolic traits (17). Our current working hypothesis is that both above-mentioned evolutionary events may be related to the adaptation of these yeasts to the floral niche. Indeed, most W/S clade species are found in association with floral nectar, pollen, and insects that visit flowers (30). In this context, the ability to assimilate sucrose is relevant because this is one of the most abundant sugars in floral nectars (31–33).

We found that the *SUC2* invertase-encoding gene of bacterial origin, used for sucrose assimilation, was almost completely restricted to the *Starmerella* subclade, the only exception being *W. spandovensis*, which may have acquired its *SUC2* gene horizontally from a *Starmerella* species. While *SUC2* presence always ensured that sucrose was metabolized (extracellularly), several *Wickerhamiella* species were found to use an alternative sucrose assimilation pathway consisting of a two-gene cluster encoding an α-glucoside transporter resembling S. cerevisiae Agt1 and an α-glucosidase that we postulate to be of the broad-substrate-range type, accepting both isomaltose- and maltose-like substrates, including sucrose (9, 10, 25, 26). The presence of this cluster correlates with sucrose assimilation in the absence of *SUC2*, with the sole exception of *W. vanderwaltii*, which is unable to use either sucrose or maltose under the conditions tested, although it possesses two apparently functional *MAL* clusters. The reasons for failure of this species to grow on disaccharides were not evident. In fact, most *Wickerhamiella* species studied in this work were unable to assimilate sucrose, while they are known to thrive in the sucrose-rich floral environment. Why some species acquired/maintained/assembled multiple strategies for sucrose assimilation while others lack these metabolic genetic tools is unclear. It has been reported that extracellular hydrolysis of complex molecules by some species can positively impact the fitness of cheater species that can make use of the public goods produced by the cooperators (34, 35). A better understanding of the composition of the microbial community of which they are part could shed some light on this topic.

Our evidence strongly supports that sucrose and maltose are the main substrates of W/S clade α-glucosidases, since the activity toward other assimilated disaccharides (melezitose and palatinose) was weak or undetectable, albeit in most cases apparently enough to support growth. This holds generally true also for Agt1 transporters. Given the niche occupied by W/S clade species, sucrose assimilation seems to be the most likely main purpose of the *MAL* pathway.

While functional characteristics of Agt transporters and α-glucosidases belonging to species in the *Wickerhamiella* and *Starmerella* subclades were very similar, the same was not true of their evolutionary histories, which appeared to be shared for each enzyme in species of the same subclade but were completely dissimilar for the same enzyme compared among subclades. The fact that in some cases the closest homologs found for the W/S clade enzymes belonged to filamentous fungi (Pezizomycotina) led us first to consider whether they might have been acquired through horizontal gene transfer, given the high frequency of this type of event in the W/S clade. However, although this cannot be completely excluded, we favor the possibility that current distribution of α-glucoside-metabolizing genes in the W/S clade reflects differential loss of ancestral gene variants.

*De novo* assembly of gene clusters, which we propose to have happened twice in the *MAL* clusters of W/S clade species, has been abundantly documented for fungi (8). Among other possible evolutionary advantages, clusters are thought to promote coexpression of genes involved in the same pathway and to facilitate gene loss and acquisition through HGT (36). Our experiments provided a glimpse into the regulation of the W/S clade MAL gene clusters, supporting the view that expression is largely constitutive, which likely reflects the absence of the transcriptional activator. In addition, there seems to be a clear absence of repression of α-glucosidase activity by glucose in at least two of the species tested. Glucose repression of the assimilation of alternative carbon sources not only is extremely common in microorganisms in general but is also known to span the entire phylogenetic range of the Saccharomycotina, since it is present in S. cerevisiae, in which it has been extensively characterized, but also in widely divergent yeast clades represented by Komagataella pastoris or Lipomyces starkeyi (37, 38). We posit that lack of glucose repression of sucrose utilization may be related to the frequent presence of both sugars simultaneously in the floral environment.

It is noteworthy that in the W/S clade the two known pathways mediating sucrose utilization in fungi are almost mutually exclusive. This dichotomy is likely due to the widely different pros and cons associated with each pathway. The first consists of the extracellular high-throughput (11) hydrolysis of sucrose by the Suc2 invertase followed by uptake of the resulting fructose and glucose by facilitated diffusion. Suc2 accepts mainly sucrose and the trisaccharide raffinose as substrates (39). Extracellular hydrolysis has important ecological repercussions because it necessarily implies some degree of sharing of sugar resources with the microbial community (40). The second pathway involves intracellular metabolism of sucrose which calls for a sugar/H^+^ symporter and a glucosidase, whose substrate range can vary considerably in yeasts but always includes sucrose and excludes raffinose (41). Hence, the Suc2 pathway requires no ATP expenditure but implies resource sharing and assimilation of a narrow range of substrates. The *MAL* pathway allows for scavenging (uptake against the concentration gradient) of a broad range of disaccharides (25), at the indirect expense of one ATP per sugar molecule for transport. We observed that *SUC2*-harboring species tend to be able to conduct alcoholic fermentation, while *MAL*-harboring species tend to be exclusively respiratory, which suggests that the preferred mode of metabolism of each species might have dictated the maintenance of energy-dependent versus energy-independent sucrose assimilation pathways. In addition, we also noted that *Wickerhamiella* species seem to have a somewhat broader niche (21), being more often isolated from substrates unrelated to the flower niche than *Starmerella* species, which might call for a broad-range pathway capable of supporting growth on a variety of disaccharides. In the W/S clade, we found only one species, *W. versatilis*, possessing (parts of) both sucrose assimilation pathways. In this species, we found an unusual extracellular α-glucosidase together with Suc2, a gene content that taken together may be assumed to support both the high sucrose throughput (11) required by fermentation and the assimilation of a broad range of disaccharides without ATP consumption. Extracellular α-glucosidases were detected in only a few fungal species (42–45); however, to our knowledge, the particular combination of tools for disaccharide utilization uncovered in *W. versatilis* was not reported before in yeasts. It seems likely that this species initially possessed a cluster similar to that found in *W. kurtzmanii* but subsequently lost the transporter after evolving an extracellular α-glucosidase. These evolutionary events could be related to the strong fermentative character of this species, since loss of the transporter ensures energetically favorable external sucrose hydrolysis via Suc2, while the extracellular glucosidase reinstates the ability to use other disaccharides that are not substrates of invertase, like maltose.

The study of different modes of sugar assimilation varying in substrate specificity and energy requirements in a phylogenetic framework is central to our understanding of the evolution of sugar metabolism but is also an important subject in yeast biotechnology (11), and the present work illustrates how the W/S clade can showcase extant natural diversity in this respect.

## MATERIALS AND METHODS

### Strains and growth conditions.

Yeast strains used in this work are listed in Table S1 with the respective source and growth conditions. For the construction of the S. cerevisiae *suc2*Δ strain, the complete coding sequence of the *SUC2* gene was eliminated by homologous recombination using a Geneticin resistance cassette. The Geneticin resistance cassette (kanR) was amplified from plasmid pWS173 (Addgene) using the set of primers indicated in Table S2.

10.1128/msphere.00035-22.9 TABLE S1 List of yeast strains used in this work for the construction of phylogenomic trees and in experimental assays. Download Table S1, PDF file, 0.8 MB. Copyright © 2022 Gonçalves et al. 2022 Gonçalves et al. https://creativecommons.org/licenses/by/4.0/ This content is distributed under the terms of the Creative Commons Attribution 4.0 International license.

10.1128/msphere.00035-22.10 TABLE S2 Primers used for heterologous expression of genes related to sucrose utilization. Download Table S2, PDF file, 0.05 MB. Copyright © 2022 Gonçalves et al. 2022 Gonçalves et al. https://creativecommons.org/licenses/by/4.0/ This content is distributed under the terms of the Creative Commons Attribution 4.0 International license.

### Identification of genes related to sucrose metabolization in W/S clade genomes.

To identify all putative α-glucosidase and α-glucoside transporters, a local query database was constructed by searching for all putative yeast α-glucosidase and α-glucoside transporters in UniProtKB (search keywords: “alpha glucosidase yeast,” “alpha glucoside transporter yeast,” and “sucrose transporter yeast”). Detailed methodology can be found in Text S1, and results are presented in Data Set S2.

10.1128/msphere.00035-22.3 TEXT S1 Detailed methodology used in this work and respective references. Download Text S1, DOCX file, 0.05 MB. Copyright © 2022 Gonçalves et al. 2022 Gonçalves et al. https://creativecommons.org/licenses/by/4.0/ This content is distributed under the terms of the Creative Commons Attribution 4.0 International license.

### Phylogenomic analysis.

Genome assemblies were obtained as described in Text S1. For the genomes sequenced in the course of this work, genomic DNA from overnight-pregrown cultures was isolated using the Quick-DNA fungal/bacterial miniprep kit (Zymo Research). Paired-end Illumina MiSeq 250-bp genomic reads were further obtained after 500 sequencing cycles at Instituto Gulbenkian Ciência. The raw sequenced reads were first preprocessed by trimming of adapters and low-quality bases using Trimmomatic v.0.33 (46). The processed reads were used to generate *de novo* assemblies using SPAdes v.3.7.0 (47), and genome assembly quality was assessed with QUAST v.4.4 (48).

For the reconstruction of the species tree (Fig. 1), single copy orthologs (SCO) were retrieved using Orthofinder 2 (49) from the predicted proteomes of W/S clade species and closest relatives (3). The resulting concatenated alignment contained 652,506 amino acid positions that were subsequently used to infer a maximum likelihood (ML) tree using IQTREE v2.0 (50) as described in Text S1. The ML phylogeny obtained using this strategy was compared with previously published and validated phylogenies using different strategies for selection of SCO (17, 30) and further validated by an additional phylogeny constructed with a different data set (Fig. S5).

10.1128/msphere.00035-22.8 FIG S5 Phylogenomic tree of two clades belonging to the Saccharomycetaceae for validation of the Orthofinder 2 strategy. (A) Species tree obtained by Shen et al. (X.X. Shen, D. A. Opulente, J. Kominek, X. Zhou, et al., Cell 175:1533–1545.e20, 2018, https://doi.org/10.1016/j.cell.2018.10.023). The internal branch that was not robustly recovered in the analyses is marked with a red circle. (B) Species tree obtained in this study using the Orthofinder 2 pipeline using Torulaspora delbrueckii and Zygosaccharomyces rouxii as outgroups. Only bootstraps below 100 are shown in the respective nodes. Sequence, alignment and tree files can be found in Figshare (https://figshare.com/articles/dataset/Phylogeny_Files/17695643). Download FIG S5, JPG file, 0.3 MB. Copyright © 2022 Gonçalves et al. 2022 Gonçalves et al. https://creativecommons.org/licenses/by/4.0/ This content is distributed under the terms of the Creative Commons Attribution 4.0 International license.

### Phylogenetic analyses of sucrose metabolism-related proteins.

To reconstruct Agt1, Ima-Mal, and Suc2 phylogenies, the closest related sequences were obtained from BLASTp searches against the UniProtKB database as described in Text S1.

For all three data sets, sequences with more than 95% similarity were removed with *CD-HIT* v.4.6.7 (51), and the remaining sequences were aligned with *MAFFT* v.7.222 (52) using an iterative refinement method (L-INS-i). Poorly aligned portions were removed with *trimAl* v.1.2 using its “gappyout” option. Phylogenetic trees were reconstructed with *IQ-TREE* v2.0 (50) using the LG+I+G4 model of substitution (found as the best-fitting model for all alignments) and ultrafast bootstrap (-bb 1,000) (53) for branch support determination. Ten independent tree searches were conducted for each data set, and the tree with the highest log likelihood was chosen. Phylogenetic trees were visualized and colored according to taxonomy using iTOL v.6 (54).

### Functional analysis of genes related to sucrose metabolism by heterologous expression in S. cerevisiae.

Plasmids p415GPD-CYC and p416TEF-CYC containing *AGT*, MAL-IMA, *SUC2*, and *SUT1*-like genes were constructed by homologous recombination in S. cerevisiae BY4741 *suc*Δ and CMY1050 *agt*Δ. The sequences of the primers used can be found in Table S3.

Recombinant S. cerevisiae strains were tested for their ability to assimilate sucrose (BY4741 *suc*Δ) and maltose (CMY1050 *agt*Δ) in test tubes as previously described.

For symport assays, the recombinant S. cerevisiae BY4741 suc^−^ strains harboring *AGT*-like homologs were pregrown in liquid 0.67% BD Difco YNB medium without amino acids with 2% (wt/vol) glucose and supplemented with uracil, methionine, and histidine. Symport assays were performed as described by Coelho et al. (14) (Text S1). Assays were performed in triplicate using two biological replicates.

### Detection of extracellular disaccharide hydrolysis.

For detection of extracellular sucrose and maltose hydrolysis, W/S clade species able to assimilate sucrose were pregrown in YP medium (1% yeast extract and 2% peptone) supplemented with 2% (wt/vol) sucrose or 2% (wt/vol) maltose until late stationary phase (when all or nearly all the sugar was consumed). Supernatants were centrifuged at maximum speed for 5 min and subsequently filtered through 0.45-μm filters. A 50-g L^−1^ concentration of sucrose or maltose was subsequently added to these cell-free supernatants. Several time points were selected to evaluate the presence of glucose (for maltose hydrolysis) or glucose and fructose (for sucrose hydrolysis) in culture supernatants, and 2-mL samples were recovered, centrifuged at 12,000 × *g* for 5 min, filtered through 0.45-μm nylon filters, and subjected to high-performance liquid chromatography (HPLC) analysis. Extracellular concentrations of sucrose, maltose, fructose, and glucose (in grams per liter) were determined by using a carbohydrate analysis column (300 mm by 7.8 mm, Aminex HPX-87P; Bio-Rad) and a differential refractometer (Shodex R-101). The column was kept at 80°C, and H_2_O was used as the mobile phase at 0.6 mL min^−1^.

### Determination of α-glucosidase activity and substrate specificity in cell extracts.

Determination of α-glucosidase activity was performed following the protocol by Viigand et al. (25). Cell-free extracts were used to measure the specific activity of hydrolysis of α-PNPG and various disaccharides. The detailed protocol can be found in Text S1, and raw data can be found in Figshare (https://figshare.com/articles/dataset/Phylogeny_Files/17695643).

### Data availability.

Genome assemblies were deposited in GenBank (BioProject PRJNA794368). Genome assemblies can be also found in Figshare (https://figshare.com/articles/dataset/Phylogeny_Files/17695643).
