# Supplementary figures and images for: Contrasting Strategies for Sucrose Utilization in a Floral Yeast Clade
Source: mSphere. 2022 Mar 31;7(2):e00035-22. doi: 10.1128/msphere.00035-22 (PMC9044934; doi:10.1128/msphere.00035-22)

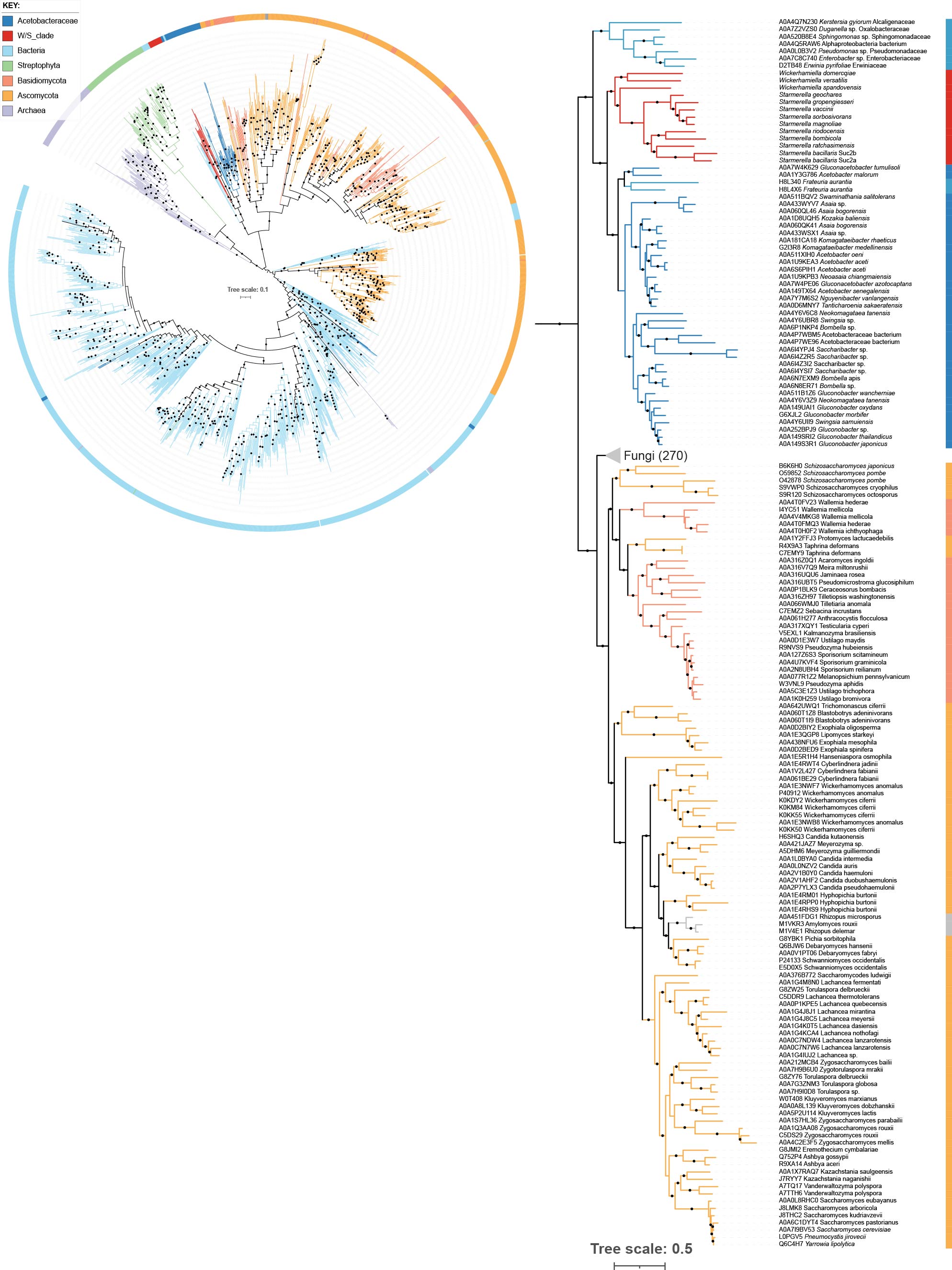

Supplement: FIG S1 [file msphere.00035-22-s0005.jpg]

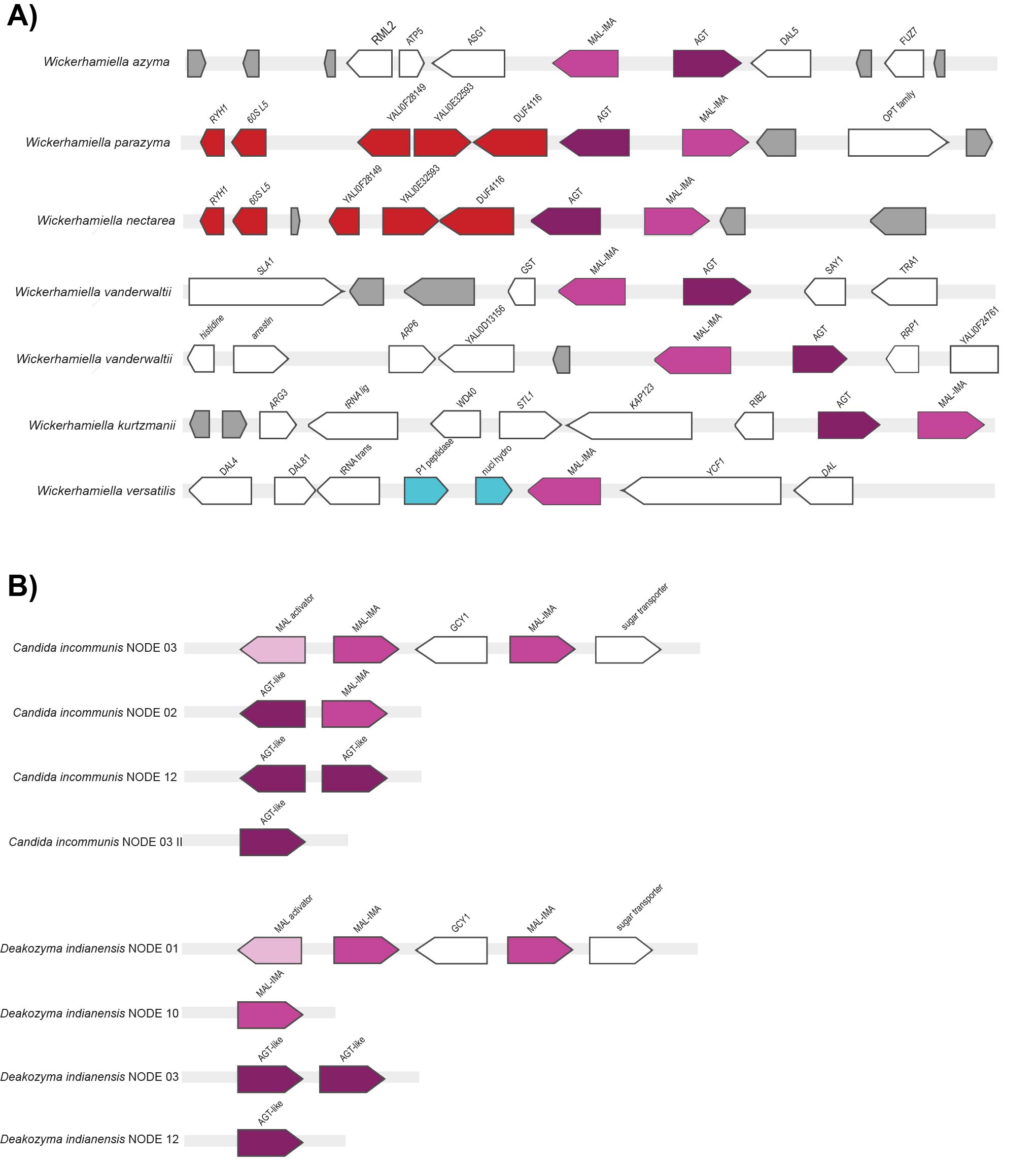

Supplement: FIG S3 [file msphere.00035-22-s0007.jpg]

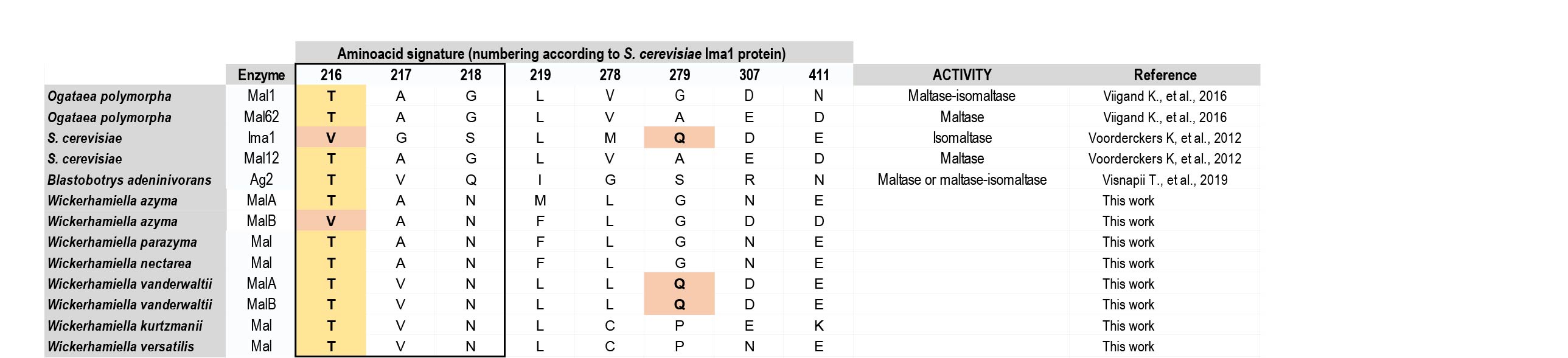

Supplement: FIG S2 [file msphere.00035-22-s0006.jpg]

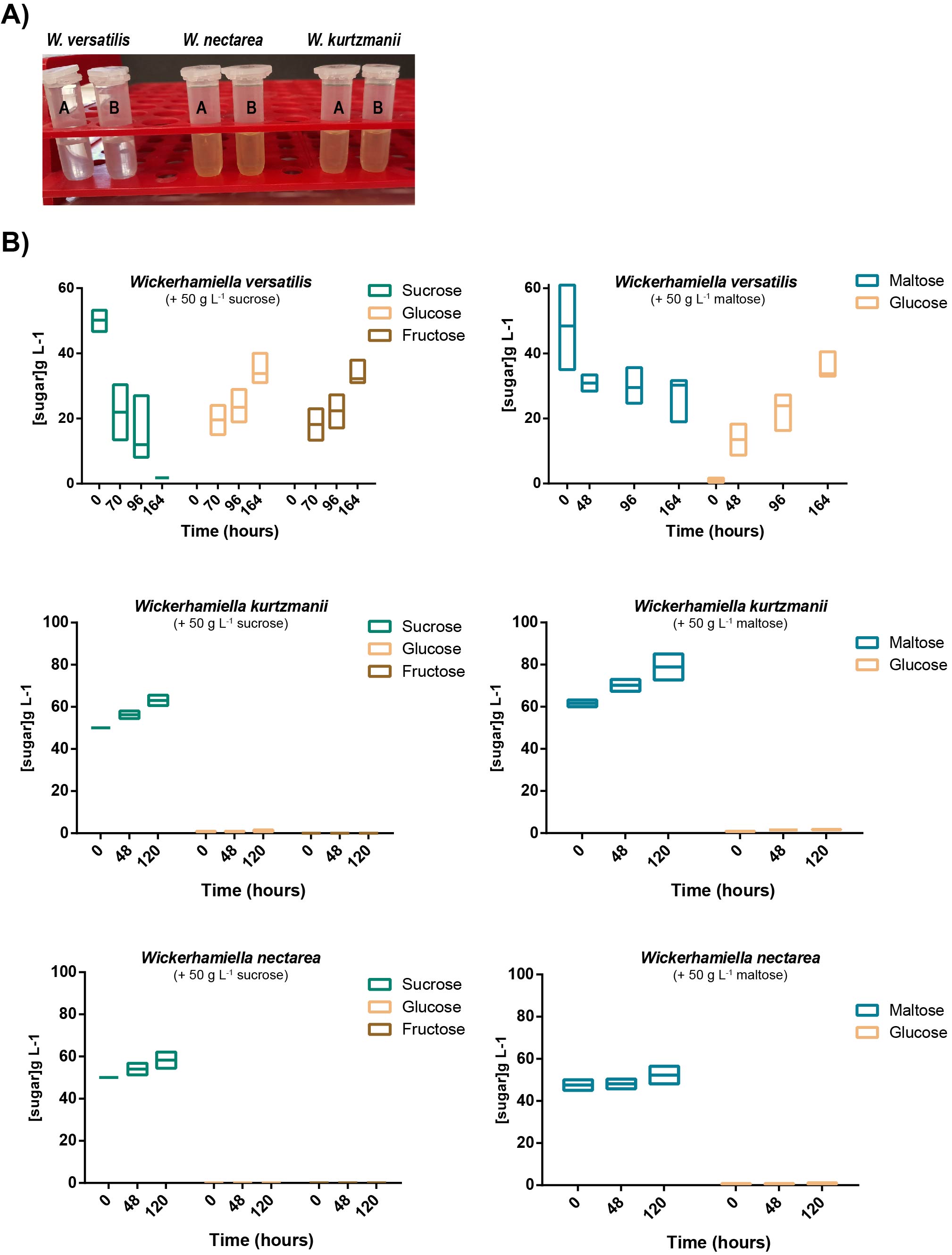

Supplement: FIG S4 [file msphere.00035-22-s0008.jpg]

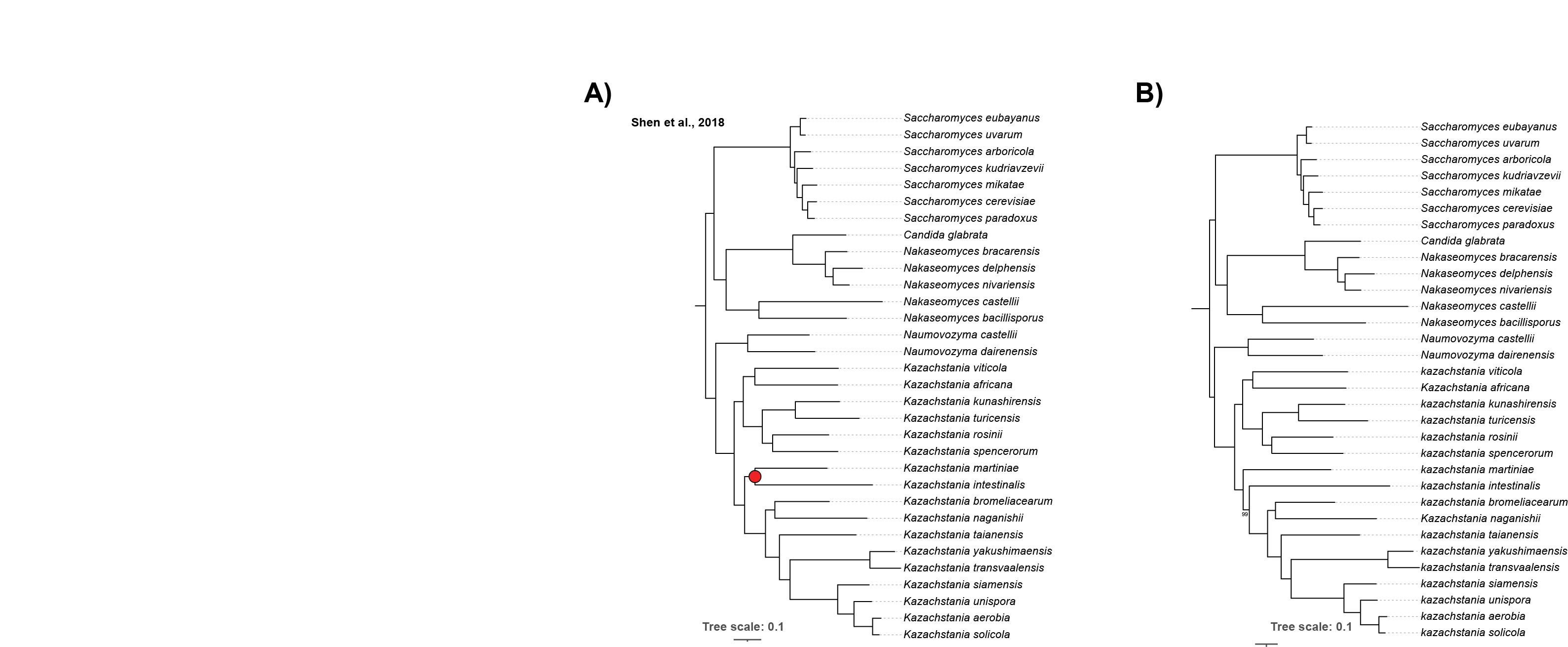

Supplement: FIG S5 [file msphere.00035-22-s0009.jpg]
