# Supplementary material for: Contrasting Strategies for Sucrose Utilization in a Floral Yeast Clade
Source: mSphere. 2022 Mar 31;7(2):e00035-22. doi: 10.1128/msphere.00035-22 (PMC9044934; doi:10.1128/msphere.00035-22)
Supplement: TABLE S1 [file msphere.00035-22-s0003.pdf]

| Species | Strain | Accession number | Phylogenetic Analyses | Reference |
|---|---|---|---|---|
| *Blastobotrys adeninivorans* | LS3 | Genolevures | Species Tree | Kunze G, et al. The complete genome of Blastobotrys (Arxula) adeninivorans LS3 - a yeast of biotechnological interest. Biotechnol Biofuels 7, 66 (2014). |
| *Blastobotrys mokoenaii* | NRRL Y-27120 | GCA_003705765.2 | Species Tree | Shen, XX et al. Tempo and Mode of Genome Evolution in the Budding Yeast Subphylum. Cell 175 (6), 1533-1545 (2018). |
| *Candida incommunis* | NRRL Y-17085 | GCA_003706695.2 | Species Tree | Shen, XX et al. Tempo and Mode of Genome Evolution in the Budding Yeast Subphylum. Cell 175 (6), 1533-1545 (2018). |
| *Deakozyma indianensis* | NRRL YB-1937 | GCA_003706415.2 | Species Tree | Shen, XX et al. Tempo and Mode of Genome Evolution in the Budding Yeast Subphylum. Cell 175 (6), 1533-1545 (2018). |
| *Nadsonia fulvescens var. fulvescens* | NRRL Y-12810 | GCA_003705595.1 | Species Tree | Shen, XX et al. Tempo and Mode of Genome Evolution in the Budding Yeast Subphylum. Cell 175 (6), 1533-1545 (2018). |
| *Saprochaete clavata (Magnusiomyces clavatus)* | CNRMA 12.647 | GCA_000817185.1 | Species Tree | Vaux S, et al. Multicenter outbreak of infections by Saprochaete clavata, an unrecognized opportunistic fungal pathogen. MBio. 2014;5(6). |
| *Starmerella bacillaris* | PYCC 3044 | GCA_003033765.1 | Species Tree | Gonçalves, C. et al. Evidence for loss and reacquisition of alcoholic fermentation in a fructophilic yeast lineage. eLife 7:e33034 (2018). |
| *Starmerella bombicola* | JCM 9596 | GCA_001599315.1 | Species Tree | Shen, XX et al. Tempo and Mode of Genome Evolution in the Budding Yeast Subphylum. Cell 175 (6), 1533-1545 (2018). |
| *Starmerella geochares* | NRRL Y-17073 | GCA_004125165.1 | Species Tree | Kominek, JZ et al. Eukaryotic Acquisition of a Bacterial Operon. Cell 176 (6), 1356-1366 (2019) |
| *Starmerella gropengiesseri* | NRRL Y-17142 | GCA_004125075.1 | Species Tree | Kominek, JZ et al. Eukaryotic Acquisition of a Bacterial Operon. Cell 176 (6), 1356-1366 (2019) |
| *Starmerella magnoliae* | PYCC 2903 | GCA_003033435.1 | Species Tree | Gonçalves, C. et al. Evidence for loss and reacquisition of alcoholic fermentation in a fructophilic yeast lineage. eLife 7:e33034 (2018). |
| *Starmerella ratchasimensis* | CBS 10611 | GCA_004124975.1 | Species Tree | Kominek, JZ et al. Eukaryotic Acquisition of a Bacterial Operon. Cell 176 (6), 1356-1366 (2019) |
| *Starmerella riodocensis* | NRRL Y-27859 | GCA_004124955.1 | Species Tree | Kominek, JZ et al. Eukaryotic Acquisition of a Bacterial Operon. Cell 176 (6), 1356-1366 (2019) |
| *Starmerella sorbosivorans* | CBS 8768 | GCA_004125005.1 | Species Tree | Kominek, JZ et al. Eukaryotic Acquisition of a Bacterial Operon. Cell 176 (6), 1356-1366 (2019) |
| *Starmerella vaccinii* | NRRL Y-17684 | GCA_004125185.1 | Species Tree | Kominek, JZ et al. Eukaryotic Acquisition of a Bacterial Operon. Cell 176 (6), 1356-1366 (2019) |
| *Sugiyamaella lignohabitans* | CBS 10342 | GCA_001640025.2 | Species Tree | Bellasio M, et al. Complete genome sequence and transcriptome regulation of the pentose utilizing yeast Sugiyamaella lignohabitans. FEMS Yeast Res. 2016 Jun;16(4). |
| *Tortispora casinolytica* | NRRL Y-17796 | GCA_001661475.1 | Species Tree | Riley R, et al. Comparative genomics of biotechnologically important yeasts. Proc Natl Acad Sci USA 113:9882-7 (2016). |
| *Wickerhamiella alocasiicola* | PYCC 8427 | PRJNA794368 | Species Tree | **This study** |
| *Wickerhamiella azyma* | PYCC 8333 | PRJNA794368 | Species Tree | **This study** |
| *Wickerhamiella cacticola* | NRRL Y-27362 | GCA_003705615.1 | Species Tree | Shen, XX et al. Tempo and Mode of Genome Evolution in the Budding Yeast Subphylum. Cell 175 (6), 1533-1545 (2018). |
| *Wickerhamiella dianesei* | PYCC 8330 | PRJNA794368 | Species Tree | **This study** |
| *Wickerhamiella domercqiae* | JCM 9478 | GCA_001599275.1 | Species Tree | Shen, XX et al. Tempo and Mode of Genome Evolution in the Budding Yeast Subphylum. Cell 175 (6), 1533-1545 (2018). |
| *Wickerhamiella galacta* | NRRL Y-17645 | GCA_003045245.1 | Species Tree | Gonçalves, C. et al. Evidence for loss and reacquisition of alcoholic fermentation in a fructophilic yeast lineage. eLife 7:e33034 (2018). |
| *Wickerhamiella hasegawae* | JCM 12559 | GCA_004125105.1 | Species Tree | Kominek, JZ et al. Eukaryotic Acquisition of a Bacterial Operon. Cell 176 (6), 1356-1366 (2019) |
| *Wickerhamiella infanticola* | NRRL Y-17858 | GCA_004125145.1 | Species Tree | Shen, XX et al. Tempo and Mode of Genome Evolution in the Budding Yeast Subphylum. Cell 175 (6), 1533-1545 (2018). |
| *Wickerhamiella kurtzmanii* | PYCC 8437 | PRJNA794368 | Species Tree | **This study** |
| *Wickerhamiella nectarea* | PYCC 8436 | PRJNA794368 | Species Tree | **This study** |
| *Wickerhamiella occidentalis* | NRRL Y-27364 | GCA_004125095.1 | Species Tree | Kominek, JZ et al. Eukaryotic Acquisition of a Bacterial Operon. Cell 176 (6), 1356-1366 (2019) |
| *Wickerhamiella pararugosa* | NRRL Y-17089 | GCA_004125235.1 | Species Tree | Kominek, JZ et al. Eukaryotic Acquisition of a Bacterial Operon. Cell 176 (6), 1356-1366 (2019) |
| *Wickerhamiella parazyma* | PYCC 8426 | PRJNA794368 | Species Tree | **This study** |
| *Wickerhamiella spandovensis* | PYCC 8431 | PRJNA794368 | Species Tree | **This study** |
| *Wickerhamiella vanderwaltii* | PYCC 3671 | PRJNA794368 | Species Tree | **This study** |
| *Wickerhamiella versatilis* | JCM 5958 | GCA_001600375.1 | Species Tree | Shen, XX et al. Tempo and Mode of Genome Evolution in the Budding Yeast Subphylum. Cell 175 (6), 1533-1545 (2018). |
| *Yarrowia lipolytica* | CLIB122 | GCA_000002525.1 | Species Tree | Dujon B, et al. Genome evolution in yeasts. Nature 430, 35–44 (2004). |
| *Nakaseomyces bracarensis* | CBS 10154 | GCA_001077315.1 | Validation Tree | Gabaldon T, et al. Comparative genomics of emerging pathogens in the Candida glabrata clade. BMC Genomics. 2013;14:623. |
| *Nakaseomyces castellii* | CBS 4332 | GCA_001046935.1 | Validation Tree | Gabaldon T, et al. Comparative genomics of emerging pathogens in the Candida glabrata clade. BMC Genomics. 2013;14:623. |
| *Candida glabrata* | CBS 138 | GCA_000002545.2 | Validation Tree | Dujon B, et al. Genome evolution in yeasts. Nature. 2004 Jul 1;430(6995):35–44. |
| *Nakaseomyces nivariensis* | CBS 9983 | GCA_017309295.1 | Validation Tree | Gabaldon T, et al. Comparative genomics of emerging pathogens in the Candida glabrata clade. BMC Genomics. 2013;14:623. |
| *Kazachstania aerobia* | NRRL Y-27976 | GCA_003708495.1 | Validation Tree | Shen, XX et al. Tempo and Mode of Genome Evolution in the Budding Yeast Subphylum. Cell 175 (6), 1533-1545 (2018). |
| *Kazachstania africana* | CBS 2517 | GCA_000304475.1 | Validation Tree | Gordon JL, et al. Evolutionary erosion of yeast sex chromosomes by mating-type switching accidents. Proc. Natl. Acad. Sci. U.S.A. 108, 20024–20029 (2011). |
| *Kazachstania bromeliacearum* | NRRL Y-48836 | GCA_003708535.2 | Validation Tree | Shen, XX et al. Tempo and Mode of Genome Evolution in the Budding Yeast Subphylum. Cell 175 (6), 1533-1545 (2018). |
| *Kazachstania intestinalis* | NRRL Y-48847 | GCA_003708845.2 | Validation Tree | Shen, XX et al. Tempo and Mode of Genome Evolution in the Budding Yeast Subphylum. Cell 175 (6), 1533-1545 (2018). |
| *Kazachstania kunashirensis* | NRRL Y-27209 | GCA_003708465.1 | Validation Tree | Shen, XX et al. Tempo and Mode of Genome Evolution in the Budding Yeast Subphylum. Cell 175 (6), 1533-1545 (2018). |
| *Kazachstania martiniae* | NRRL Y-40 | GCA_003708925.2 | Validation Tree | Shen, XX et al. Tempo and Mode of Genome Evolution in the Budding Yeast Subphylum. Cell 175 (6), 1533-1545 (2018). |
| *Kazachstania naganishii* | CBS 8797 | GCA_000348985.1 | Validation Tree | Gordon JL, et al. Evolutionary erosion of yeast sex chromosomes by mating-type switching accidents. Proc. Natl. Acad. Sci. U.S.A. 108, 20024–20029 (2011). |
| *Kazachstania rosinii* | NRRL Y-17919 | GCA_003708425.2 | Validation Tree | Shen, XX et al. Tempo and Mode of Genome Evolution in the Budding Yeast Subphylum. Cell 175 (6), 1533-1545 (2018). |
| *Kazachstania siamensis* | NRRL Y-48842 | GCA_003708905.2 | Validation Tree | Shen, XX et al. Tempo and Mode of Genome Evolution in the Budding Yeast Subphylum. Cell 175 (6), 1533-1545 (2018). |
| *Kazachstania solicola* | NRRL Y-27207 | GCA_003708835.2 | Validation Tree | Shen, XX et al. Tempo and Mode of Genome Evolution in the Budding Yeast Subphylum. Cell 175 (6), 1533-1545 (2018). |
| *Kazachstania spencerorum* | NRRL Y-17920 | GCA_003708825.2 | Validation Tree | Shen, XX et al. Tempo and Mode of Genome Evolution in the Budding Yeast Subphylum. Cell 175 (6), 1533-1545 (2018). |
| *Kazachstania taianensis* | NRRL Y-48846 | GCA_003708865.1 | Validation Tree | Shen, XX et al. Tempo and Mode of Genome Evolution in the Budding Yeast Subphylum. Cell 175 (6), 1533-1545 (2018). |
| *Kazachstania transvaalensis* | NRRL Y-17245 | GCA_003708445.2 | Validation Tree | Shen, XX et al. Tempo and Mode of Genome Evolution in the Budding Yeast Subphylum. Cell 175 (6), 1533-1545 (2018). |
| *Kazachstania turicensis* | NRRL Y-48834 | GCA_003708545.1 | Validation Tree | Shen, XX et al. Tempo and Mode of Genome Evolution in the Budding Yeast Subphylum. Cell 175 (6), 1533-1545 (2018). |
| *Kazachstania unispora* | NRRL Y-1556 | GCA_003708525.2 | Validation Tree | Shen, XX et al. Tempo and Mode of Genome Evolution in the Budding Yeast Subphylum. Cell 175 (6), 1533-1545 (2018). |
| *Kazachstania viticola* | NRRL Y-27206 | GCA_003708455.1 | Validation Tree | Shen, XX et al. Tempo and Mode of Genome Evolution in the Budding Yeast Subphylum. Cell 175 (6), 1533-1545 (2018). |
| *Kazachstania yakushimaensis* | NRRL Y-48837 | GCA_003709265.1 | Validation Tree | Shen, XX et al. Tempo and Mode of Genome Evolution in the Budding Yeast Subphylum. Cell 175 (6), 1533-1545 (2018). |
| *Nakaseomyces bacillisporus* | CBS 7720 | GCA_001046975.1 | Validation Tree | Gabaldon T, et al. Comparative genomics of emerging pathogens in the Candida glabrata clade. BMC Genomics. 2013;14:623. |
| *Nakaseomyces delphensis* | CBS 2170 | GCA_001039675.1 | Validation Tree | Gabaldon T, et al. Comparative genomics of emerging pathogens in the Candida glabrata clade. BMC Genomics. 2013;14:623. |
| *Naumovozyma castellii* | CBS 4309 | GCA_000237345.1 | Validation Tree | Gordon JL, et al. Evolutionary erosion of yeast sex chromosomes by mating-type switching accidents. Proc. Natl. Acad. Sci. U.S.A. 108, 20024–20029 (2011). |
| *Naumovozyma dairenensis* | CBS 421 | GCA_000227115.2 | Validation Tree | Gordon JL, et al. Evolutionary erosion of yeast sex chromosomes by mating-type switching accidents. Proc. Natl. Acad. Sci. U.S.A. 108, 20024–20029 (2011). |
| *Saccharomyces paradoxus* | CBS 432 | GCA_002079055.1 | Validation Tree | Liti G, et al. Population genomics of domestic and wild yeasts. Nature. 2009 Mar 19;458(7236):337–41. |
| *Saccharomyces arboricola* | CBS 10644 | GCA_000292725.1 | Validation Tree | Liti G, et al. High quality de novo sequencing and assembly of the Saccharomyces arboricolus genome. BMC Genomics 14, 69 (2013). |
| *Saccharomyces cerevisiae* | S288C | SGD | Validation Tree | Goffeau A, et al. Life with 6000 genes. Science 274, 546, 563–567 (1996). |
| *Saccharomyces eubayanus* | FM1318 | GCA_001298625.1 | Validation Tree | Baker E, et al. The Genome Sequence of Saccharomyces eubayanus and the Domestication of Lager-Brewing Yeasts. Mol Biol Evol 32, 2818–2831 (2015). |
| *Saccharomyces kudriavzevii* | IFO 1802 | GCA_000167075.2 | Validation Tree | Scannell DR, et al. The Awesome Power of Yeast Evolutionary Genetics: New Genome Sequences and Strain Resources for the Saccharomyces sensu stricto Genus. G3 (Bethesda) 1, 11–25 (2011). |
| *Saccharomyces mikatae* | IFO 1815 | GCA_000166975.1 | Validation Tree | Scannell DR, et al. The Awesome Power of Yeast Evolutionary Genetics: New Genome Sequences and Strain Resources for the Saccharomyces sensu stricto Genus. G3 (Bethesda) 1, 11–25 (2011). |
| *Saccharomyces uvarum* | CBS 7001 | GCA_019953615.1 | Validation Tree | Scannell DR, et al. The Awesome Power of Yeast Evolutionary Genetics: New Genome Sequences and Strain Resources for the Saccharomyces sensu stricto Genus. G3 (Bethesda) 1, 11–25 (2011). |
| *Torulaspora delbrueckii* | CBS 1146 | GCA_000243375.1 | Validation Tree | Gordon JL, et al. Evolutionary erosion of yeast sex chromosomes by mating-type switching accidents. Proc. Natl. Acad. Sci. U.S.A. 108, 20024–20029 (2011). |
| *Zygosaccharomyces rouxii* | CBS 732 | GCA_000026365.1 | Validation Tree | Génolevures Consortium, et al. Comparative genomics of protoploid Saccharomycetaceae. Genome Res. 19, 1696–1709 (2009). |
