# Supplementary material for: Contrasting Strategies for Sucrose Utilization in a Floral Yeast Clade
Source: mSphere. 2022 Mar 31;7(2):e00035-22. doi: 10.1128/msphere.00035-22 (PMC9044934; doi:10.1128/msphere.00035-22)
Supplement: TABLE S2 [file msphere.00035-22-s0004.pdf]

Primer Name	Primer Sequence (5’ – 3’)	Purpose
SUC2_kanRLong_Fw	AAAACAAAAAGCTTTTCTTTTCACTAACGTATAT GAGCTTGCCTCGTCCCCG	Deletion of *SUC2* in *S. cerevisiae* BY4741 strain using a kanR resistance cassette
SUC2_kanRLong_Rv	GAAAAAAATAAAAAAGACAATAAGTTTTATAAC CTCAGTATAGCGACCAGCATTCAC	
Wpara_p416TEF_IMA_Fw	AGCATAGCAATCTAATCTAAGTTTTAATTACAA ATCTAGAATGAAAGGACAGTGGTGG	Heterologous expression of *MAL-IMA* from *W. parazyma* in p416TEFCYC in *S. cerevisiae*
Wpara_p416TEF_IMA_Rv	CGTGAATGTAAGCGTGACATAACTAATTACATG ACTCGAGCTAAGCGTAGACGCGGG	
Wnect_p416TEF_IMA_Fw	AGCATAGCAATCTAATCTAAGTTTTAATTACAA ATCTAGAATGAGTGGACAGTGGTG	Heterologous expression of *MAL-IMA* from *W. nectarea* in p416TEFCYC in *S. cerevisiae*
Wnect_p416TEF_IMA_Rv	CGTGAATGTAAGCGTGACATAACTAATTACATG ACTCGAGCTACGCGTAAATGCGAG	
Wkurt_p416TEF_IMA_Fw	AGCATAGCAATCTAATCTAAGTTTTAATTACAA ATCTAGAATGGTCACCAAAGCTTGG	Heterologous expression of *MAL-IMA* from *W. kurtzmanii* in p416TEFCYC in *S. cerevisiae*
WKurt_p416TEF_IMA_Rv	CGTGAATGTAAGCGTGACATAACTAATTACATG ACTCGAGTTACTTTTGGCACAGGTATAC	
Stbomb_p416TEF_SUC2_Fw	AGCATAGCAATCTAATCTAAGTTTTAATTACAA ATCTAGAATGTCCGAAGAAGAAATGTC	Heterologous expression of *SUC2* from *St. bombicola* in p416TEFCYC in *S. cerevisiae*
Stbomb_p416TEF_SUC2_Rv	CGTGAATGTAAGCGTGACATAACTAATTACATG ACTCGAGTCAAGGAAGAGTTGCTCTC	
Wspand_p416TEF_SUC2_Fw	AGCATAGCAATCTAATCTAAGTTTTAATTACAA ATCTAGAATGACCGGCCTCCCACG	Heterologous expression of *SUC2* from *W. spandovensis* in p416TEFCYC in *S. cerevisiae*
Wspand_p416TEF_SUC2_Rv	TGAATGTAAGCGTGACATAACTAATTACATGAC TCGAGCTAAGCAAGGGTCTCACGGTTC	
Wpara_p416GPD_AGT_Fw	AGTTTTAAAACACCAGAACTTAGTTTCGACGGA TTCTAGAATGTCTATCAGGTCTCTCAA	Heterologous expression of *AGT* from *W. parazyma* in p416TEFCYC in *S. cerevisiae*
Wpara_p415GPD_AGT_Rv	CGTGAATGTAAGCGTGACATAACTAATTACATG ACTCGAGTCAAGCCAGCTCAACATG	
Wnect_p415GPD_AGT_Fw	AGTTTTAAAACACCAGAACTTAGTTTCGACGGA TTCTAGAATGTCTCTGAGGTCTCTAA	Heterologous expression of *AGT* from *W. nectarea* in p416TEFCYC in *S. cerevisiae*
Wnect_p415GPD_AGT_Rv	CGTGAATGTAAGCGTGACATAACTAATTACATG ACTCGAGCTAAGCAATCTCGACGTG	
Wkurtz_p415GPD_AGT_Fw	AGTTTTAAAACACCAGAACTTAGTTTCGACGGA TTCTAGAATGACCCAAGAAATGGTATC	Heterologous expression of *AGT* from *W. kurtzmanii* in p416TEFCYC in *S. cerevisiae*
Wkurtz_p415GPD_AGT_Rv	CGTGAATGTAAGCGTGACATAACTAATTACATG ACTCGAGTTATGGCTGTTTCAGGTATTC	
Wversa_p415GPD_SUT1_Fw	AGTTTTAAAACACCAGAACTTAGTTTCGACGGA TTCTAGAATGGAGTGGGTAGGGAT	Heterologous expression of *SUT1-like* from *W. versatilis* in p416TEFCYC in *S. cerevisiae*
Wversa_p415GPD_SUT1_Rv	CGTGAATGTAAGCGTGACATAACTAATTACATG ACTCGAGTCAATGAACGCGAATCTTAC	
