# Supplementary material for: Contrasting Strategies for Sucrose Utilization in a Floral Yeast Clade
Source: mSphere. 2022 Mar 31;7(2):e00035-22. doi: 10.1128/msphere.00035-22 (PMC9044934; doi:10.1128/msphere.00035-22)
Supplement: TEXT S1 [file msphere.00035-22-s0010.docx]

**Supplemental methods**

**Strains and growth conditions**

The *Wickerhamiella* and *Starmerella* strains used in this work are listed in Table S1 (Figshare: 10.6084/m9.figshare.17695643) and were obtained from the Portuguese Yeast Culture Collection (PYCC, Portugal). The strains used for phenotypic assays were the same for which genome sequences were obtained. All strains were maintained in YMA medium [0.3% (w/v) yeast extract, 0.3% (w/v) malt extract, 0.5% (w/v) bacto-peptone and 1% (w/v) glucose]. Two *Saccharomyces* *cerevisiae* laboratory strains were used for heterologous expression to evaluate the ability of the candidate genes to support growth on sucrose (*S. cerevisiae* BY4741: *MATa his3Δ leu2Δ met15Δ* *ura3Δ*) or on maltose (*S. cerevisiae* CMY1050/PYCC5650: *MATa mal11::HIS3 MAL12 MAL13 leu2 ura3-52 lys2-801 ade2-101 trp1-63*) (1). To abolish sucrose assimilation in the BY4741 strain, the complete coding sequence of the *SUC2* gene was eliminated by homologous recombination using a geneticin resistance cassette. The geneticin resistance cassette (kanR) was amplified from plasmid pWS173 (Addgene) using the set of primers listed in Table S2.

**Assimilation of several α-glucosides by W/S-clade species**

All the strains were tested for their ability to assimilate sucrose and maltose. For that, a cell-suspension (OD_640nm_ ~0.8) was prepared in sterile water and subsequently used to inoculate (100 μL) test tubes containing 5 mL of Yeast Nitrogen Base (YNB) medium supplemented with 2% (w/v) glucose, 2% (w/v) sucrose or 2% (w/v) maltose. Unsupplemented YNB was used as the negative control. The cultures were grown for up to 10 days at 25ºC with orbital shaking. For *AGT*-*MAL* containing species, the ability to assimilate other maltose or isomaltose substrates (melezitose and palatinose) was also tested in the same way. Assays were performed independently in triplicate.

**Identification of genes related to sucrose metabolization in W/S-clade genomes**

Identification of *SUC2* genes was performed as in Gonçalves et al., 2018 (2). For each Suc2 sequence, *in silico* prediction of the subcellular localization was performed by submitting the sequences into SignalP (3) and DeepLoc (4) softwares. The results are presented in Data Set S1. To identify all putative α-glucosidase and α-glucoside transporters, a local query database was constructed by searching for all putative yeast α-glucosidase and α-glucoside transporters in UniprotKB (search keywords: "alpha glucosidase yeast", "alpha glucoside transporter yeast" and "sucrose transporter yeast"). A local BLASTp search was conducted using all putative α-glucosidase sequences (total of 1.113) and all putative sucrose/α-glucoside transporter sequences (total of 1.398) against a local proteome database constructed with the predicted proteomes from all W/S-clade species and outgroups *Candida incommunis* and *Blastobotrys adeninivorans.* The complete proteome for each species was predicted with AUGUSTUS (5) using the complete model and *S. cerevisiae* as reference as in Gonçalves et al., 2018. For *W. domercqiae* JCM 9478*, W. versatilis* JCM 5958, publicly available proteomes were used. An e-value cut-off of 1e^-3^ was set, and the resulting hits were subsequently used in a BLASTp search against the NCBI non-redundant (nr) database to evaluate whether they corresponded to putative α-glucosidases/α-glucoside transporters. The results are presented in Data Set S2. Presence and absence of the flagged genes (*SUC2*, *IMA*-*MAL*, *AGT*) was reconfirmed by tBLASTn (e-value cutoff e^-3^) against a local database containing the 25 W/S-clade genomes inspected and the genomes of closest relatives *Candida* *incommunis* and *Deakozyma* *indianensis*. Hits contained in small (< 500 bp) and low coverage (< 1x) contigs were discarded. For Ima-Mal and Agt hits, *e*-values > e^-50^ corresponded to other unrelated protein families. The raw BLAST results can be found in Figshare (DOI: 10.6084/m9.figshare.17695643).

**Phylogenomic analysis**

Genome assemblies from W/S-clade species *W. infanticola*, *W. hasegawae*, *W. galacta*, *W. pararugosa*, *W. cacticola*, *W. occidentalis*, *W. versatilis*, *W. domercqiae*, *St. ratchasimensis*, *St. riodocensis*, *St. gropengiesserii*, *St. vaccinii*, *St. magnoliae*, *St. bombicola*, *St. geochares* and *St. sorbosivorans* and closest related species *Candida* *incommunis*, *Deakozyma* *indianensis*, *Blastrobotrys* *adeninivorans*, *Blastrobotrys* *mokoenaii*, *Nadsonia* *fulvescens*, *Magnusiomyces clavatus* (6), *Yarrowia* *lipolytica*, *Sugiyamaella* *lignohabitans* and *Tortispora* *caseinolytica* were obtained from NCBI (2, 7, 8). The remaining genomes were sequenced in the course of this work: *W. spandovensis*, *W. azyma*, *W. parazyma*, *W. nectarea*, *W. vanderwaltii*, *W. alocasiicola*, *W. dianesei* and *W. kurtzmanii*. For each species, genomic DNA from overnight grown cultures was isolated using the Quick-DNA Fungal/Bacterial Miniprep kit (Zymoresearch). Paired-end Illumina MiSeq 250 bp genomic reads were further obtained after 500 sequencing cycles at Instituto Gulbenkian Ciência Oeiras, Portugal). The raw sequenced reads were first pre-processed by trimming of adapters and low-quality bases using Trimmomatic v0.33 (9). The processed reads were used to generate *de novo* assemblies using SPAdes v3.7.0 (10) and genome assembly quality was assessed with QUAST v4.4 (11).

Genome assemblies were deposited at GenBank under the accession PRJNA794368 and can be also accessed in Figshare (DOI: 10.6084/m9.figshare.17695643).

For the reconstruction of the species tree (Fig. 1), single copy orthologs (SCO) were retrieved using Orthofinder 2 (12) from the predicted proteomes of W/S-clade species and closest relatives (obtained as previously described). The resulting concatenated alignment contained 652.506 aminoacid positions that were subsequently used to infer a Maximum Likelihood (ML) tree using IQTREE v2.0 (13) with an automatic detection of the best-fitting model of amino acid evolution and 1.000 ultrafast bootstrapping replicates (14). A total of five independent tree searches were conducted (--runs 5) and the tree with the highest likelihood score was selected as the one representing the most likely phylogenetic relationships between species. A total number of 1.200 orthogroups were used to construct the species tree alignment in Orthofinder 2 (list of orthogoups and respective sequences are listed in Figshare: 10.6084/m9.figshare.17695643). The phylogeny was rooted using *Tortispora* *caseinolytica* as the outgroup based on the phylogenetic analyses of Shen et al., 2018. The obtained ML phylogeny using this strategy was compared with previously published and validated phylogenies using different strategies for selection of SCO (7, 15). To further validate the Orthofinder 2 strategy, an additional phylogeny was constructed using a different dataset composed of two clades belonging to the Saccharomycetaceae (*Saccharomyces* and *Kazachstania* clades) used in Shen et al. 2018. Proteomes were predicted as described above and the results are presented in Fig. S5. The list and sequences of orthogroups, alignment sequences, and tree files can be found in Figshare (DOI: 10.6084/m9.figshare.17695643). All the relationships between the species could be reproduced except for the internal branch grouping *Kazachstania intestinalis* and *Kazachstania martiniae* that was one of the branches also not robustly recovered in the analyses by Shen et al., 2018.

**Phylogenetic analyses of sucrose metabolism-related proteins**

To reconstruct the Suc2 phylogeny, the Suc2 protein sequence from *St. bombicola* was used in five independent BLASTp searches against the UniProtKB database: UniProtKB (1.000 top hits), UniProtKB_Fungi (top 1.000 hits), UniProtKB_Bacteria (top 1.000 hits), UniProtKB_Plants (top 100 hits) and UniProtKB_Archaea (top 100 hits), so as to include invertase sequences from major eukaryotic and prokaryotic lineages.

To reconstruct the Mal-Ima protein phylogeny, the top 1.000 top hits were retrieved from UniProtKB using Ima1 from *S. cerevisiae*, the Mal-Ima protein from *W. azyma* and one Mal-Ima protein from *C. incommunis* as queries in three independent BLASTp searches.

To reconstruct the Agt-like phylogeny, the top 1.000 hits were retrieved from UniProtKB using Agt1 from *S. cerevisiae*, Agt-like protein from *W. azyma* and Agt-like protein from C*. incommunis* as queries in three independent BLASTp searches. Given that preliminary Mal-Ima phylogenies suggested that W/S-clade sequences clustered with those from *B. adeninivorans*, all Agt-like sequences from this species were retrieved (Data Set S2) and added to the dataset.

For all three datasets, sequences with more than 95% similarity were removed with *CD-HIT* v4.6.7 (16) and the remaining sequences were aligned with *MAFFT* v7.222 (17) using an iterative refinement method (L-INS-i). Poorly aligned portions were removed with *trimAl* v1.2 using its “gappyout” option. Phylogenetic trees were reconstructed with *IQ-TREE* v2.0 (13) using the LG+I+G4 model of substitution (found as the best fitting model for all alignments) and ultrafast bootstrap (-bb 1.000) for branch support determination. Ten independent tree searches were conducted for each dataset and the tree with the highest log-likelihood was chosen. Phylogenetic trees were visualized and colored according to taxonomy using iTOL v6 (18).

**Heterologous expression of candidate genes related to sucrose metabolism in *S. cerevisiae***

Plasmids p415GPD-CYC carrying *AGT*-like genes (from *W. parazyma*, *W. nectarea* and *W. kurtzmanii*) and plasmids P416TEF-CYC carrying *SUC2* (from *W. spandovensis* and *St. bombicola*) and *MAL*-*IMA* genes (from *W. parazyma*, *W. nectarea* and *W. kurtzmanii*) were constructed by homologous recombination in *S. cerevisiae* BY4741 *suc2Δ*. Plasmids P415GPD-CYC containing the putative *SUT1*-like gene from *W. versatilis* and the *AGT*-like genes were also constructed by homologous recombination in *S. cerevisiae* CMY1050 (PYCC 5650). The sequences of the primers used can be found in Table S2. All fragments were amplified with Phusion High Fidelity (Thermo Fisher Scientific).

**Growth and symport assays**

For growth assays, recombinant *S. cerevisiae* strains harboring *AGT*, *MAL-IMA*, *AGT*+*MAL*-*IMA* and *SUT1*-like homologs were tested for their ability to assimilate sucrose (BY4741 *suc2Δ*) and maltose (*agtΔ*, CMY1050) (1) in test tubes as described in previous section “Assimilation of several α-glucosides by W/S-clade species “ but using YNB medium without amino acids instead, supplemented with the appropriate set of amino acids for each recombinant strain. Assays were performed in triplicate using two biological replicates.

For symport assays, the recombinant *S. cerevisiae* BY4741 *suc2Δ* strains harboring *AGT*-like homologs were grown in liquid 0.67% BD Difco YNB medium without aminoacids with 2% (w/v) of glucose and supplemented with uracil, methionine, and histidine. The cultures were grown until mid-exponential phase (OD_640nm_ between 0.8 and 1.2), harvested by centrifugation at 4ºC, washed twice with ice-cold sterile water and resuspended in water to a final concentration of 30-40 mg dry weight/mL. Presence of symport activity was assessed by recording the alkalization (using a standard pH meter) of an aqueous cell suspension during 120 seconds after the addition of 10 mM of sucrose, maltose or melezitose. Viability of cell transport was assayed by adding 10 mM of glucose to the cell suspension and observation of acidification. Cells from the *S. cerevisiae* recombinant strain harboring the *AGT* gene from *W. kurtzmanii* did not show symport activity when pre-grown on glucose but activity was recorded when cells were pre-grown on 0.2% (w/v) of glucose and 1.8% (w/v) of sucrose. *Saccharomyces cerevisiae* strains harboring empty p415GPD-CYC plasmids was used as the negative control.

All assays were performed in triplicate but only one representative assay is shown in Fig. 3.

**Detection of extracellular disaccharide hydrolysis**

For detection of extracellular sucrose hydrolysis, two distinct assays were performed. First, W/S-clade species able to assimilate sucrose were grown in YP medium (1% yeast extract and 2% peptone) supplemented with 20% (w/v) of sucrose. Three distinct time points were selected to evaluate the presence of glucose and fructose in culture supernatants and 2 mL samples were recovered, centrifuged at 12,000 x g for 5 min, filtered through 0.45μm filters and maintained at -20ºC until submitted to HPLC analysis. Secondly, detection of extracellular invertase activity was also performed by growing the cultures in YP medium supplemented with 2% (w/v) of sucrose until late stationary phase (when all or nearly all the sugar was consumed). Supernatants were centrifuged at maximum speed for 5 minutes and subsequently filtered through 0.45 μm filters. 50 g L^-1^ of sucrose were subsequently added to these cell free supernatants and samples were taken at several time points. Samples were treated as previously described for HPLC analyses. Extracellular concentrations of sucrose, fructose and glucose (g L^−1^) were determined by using a carbohydrate analysis column (300 mm × 7.8 mm, Aminex HPX-87P; Biorad) and a differential refractometer (Shodex R-101). The column was kept at 80 °C and H_2_O was used as the mobile phase at 0.6 mL min^−1^. For detection of extracellular ɑ-glucosidase activity, the same strategy was followed but cells were pre-grown in YP medium supplemented with 2% (w/v) of maltose instead and 50 g L^-1^ of maltose was added to the filtered supernatant.

**Determination of ɑ-glucosidase activity and substrate specificity in cell-free extracts**

Determination of ɑ-glucosidase activity was performed following the protocol by Viigand K., et al., 2016 (19). Briefly, cells were pre-grown in 0.67% (w/v) BD Difco YNB medium supplemented with 0.2% (w/v) glucose at 25ºC with orbital shaking. The cultures were subsequently re-inoculated in YNB medium supplemented with 2% (w/v) of each sugar: glucose, sucrose, maltose, or glycerol and grown until early/mid-exponential phase (OD_640nm_ ~ 1 – 3). Prior to centrifugation, glucose concentration was checked for each culture growing in YNB + 2% glucose using Combur Test (Roche) to make sure > 5 g L^-1^ were still present (repressing conditions). The cells were collected by centrifugation at 4ºC, washed twice with cold maltase buffer [100 mM potassium phosphate buffer with 0.1 mM ethylenediaminetetraacetic acid (EDTA), pH 6.5], resuspended in maltase buffer and disrupted using glass-beads in six 30-second cycles of vortex/ice. The total protein concentration in the extracts was quantified using Qubit 2.0. Cell-free extracts were used to measure the specific activity of hydrolysis of α-pNPG and various disaccharides.

The cell extracts were assayed for the hydrolysis of 1 mM α-pNPG in maltase buffer at 37ºC. Initial velocity of the reaction was measured by recording *p*-nitrophenol release (absorbance at 405 nm) using 40 μg of total protein in a final volume of 1 mL. At fixed time points (up to 15 minutes), aliquots were combined with three volumes of 200 mM TRIS buffer (pH 8.3) and subsequently heated at 96ºC for 5 minutes to inactivate enzyme activity. At least three independent experiments were performed and for each assay two independent measurements were performed. *Wickerhamiella* *alocasiicola* cell extracts were used as a negative control because this species lacks *MAL*-*IMA* genes and *Saccharomyces* *cerevisiae* was used as a positive control. Relative activity was calculated for each condition by comparing each of the slopes (obtained from linear regression on the exponential part of the curve) with those obtained for maltose in each assay. Relative activity was calculated by dividing each of the slopes (obtained by plotting the OD_405nm_ against time) by the slope obtained for the condition where cells were grown in 2% (w/v) maltose. The substrate specificity of Mal proteins was determined by measuring the release of glucose when cell-free extracts (20 μg of total protein in 100 μL of final reaction volume) were incubated at 37ºC for 10 minutes with 250 mM of each glucoside: sucrose, maltose, melezitose, or palatinose. The release of glucose was determined spectrophotometrically using the D-Glucose GOD-POD kit (NZYtech) and calculated according to the manufacturer’s instructions. Raw data can be found in Figshare: 10.6084/m9.figshare.17695643.

**Supplemental References**

1. Salema-Oom M, Valadão Pinto V, Gonçalves P, Spencer-Martins I. 2005. Maltotriose utilization by industrial *Saccharomyces* strains: characterization of a new member of the alpha-glucoside transporter family. Appl Environ Microbiol 71:5044-9.

2. Gonçalves C, Wisecaver JH, Kominek J, Oom MS, Leandro MJ, Shen X-X, Opulente DA, Zhou X, Peris D, Kurtzman CP, Hittinger CT, Rokas A, Gonçalves P. 2018. Evidence for loss and reacquisition of alcoholic fermentation in a fructophilic yeast lineage. eLife 7:e33034.

3. Almagro Armenteros JJ, Tsirigos KD, Sønderby CK, Petersen TN, Winther O, Brunak S, von Heijne G, Nielsen H. 2019. SignalP 5.0 improves signal peptide predictions using deep neural networks. Nature Biotechnology 37:420-423.

4. Almagro Armenteros JJ, Sønderby CK, Sønderby SK, Nielsen H, Winther O. 2017. DeepLoc: prediction of protein subcellular localization using deep learning. Bioinformatics 33:3387-3395.

5. Stanke M, Keller O, Gunduz I, Hayes A, Waack S, Morgenstern B. 2006. AUGUSTUS: ab initio prediction of alternative transcripts. Nucleic acids research 34:W435-W439.

6. Schoch CL, Ciufo S, Domrachev M, Hotton CL, Kannan S, Khovanskaya R, Leipe D, McVeigh R, O’Neill K, Robbertse B, Sharma S, Soussov V, Sullivan JP, Sun L, Turner S, Karsch-Mizrachi I. 2020. NCBI Taxonomy: a comprehensive update on curation, resources and tools. Database 2020.

7. Shen XX, Opulente DA, Kominek J, Zhou X, Steenwyk JL, Buh KV, Haase MAB, Wisecaver JH, Wang M, Doering DT, Boudouris JT, Schneider RM, Langdon QK, Ohkuma M, Endoh R, Takashima M, Manabe RI, Cadez N, Libkind D, Rosa CA, DeVirgilio J, Hulfachor AB, Groenewald M, Kurtzman CP, Hittinger CT, Rokas A. 2018. Tempo and Mode of Genome Evolution in the Budding Yeast Subphylum. Cell 175:1533-1545.e20.

8. Krause DJ, Kominek J, Opulente DA, Shen XX, Zhou X, Langdon QK, DeVirgilio J, Hulfachor AB, Kurtzman CP, Rokas A, Hittinger CT. 2018. Functional and evolutionary characterization of a secondary metabolite gene cluster in budding yeasts. Proc Natl Acad Sci U S A 115:11030-11035.

9. Bolger AM, Lohse M, Usadel B. 2014. Trimmomatic: a flexible trimmer for Illumina sequence data. Bioinformatics 30:2114-2120.

10. Bankevich A, Nurk S, Antipov D, Gurevich AA, Dvorkin M, Kulikov AS, Lesin VM, Nikolenko SI, Pham S, Prjibelski AD, Pyshkin AV, Sirotkin AV, Vyahhi N, Tesler G, Alekseyev MA, Pevzner PA. 2012. SPAdes: a new genome assembly algorithm and its applications to single-cell sequencing. Journal of computational biology : a journal of computational molecular cell biology 19:455-477.

11. Gurevich A, Saveliev V, Vyahhi N, Tesler G. 2013. QUAST: quality assessment tool for genome assemblies. Bioinformatics 29:1072-1075.

12. Emms DM, Kelly S. 2019. OrthoFinder: phylogenetic orthology inference for comparative genomics. Genome Biology 20:238.

13. Nguyen LT, Schmidt HA, von Haeseler A, Minh BQ. 2015. IQ-TREE: a fast and effective stochastic algorithm for estimating maximum-likelihood phylogenies. Mol Biol Evol 32:268-74.

14. Hoang DT, Chernomor O, von Haeseler A, Minh BQ, Vinh LS. 2018. UFBoot2: Improving the Ultrafast Bootstrap Approximation. Mol Biol Evol 35:518-522.

15. Gonçalves P, Gonçalves C, Brito PH, Sampaio JP. 2020. The *Wickerhamiella*/*Starmerella* clade—A treasure trove for the study of the evolution of yeast metabolism. Yeast n/a.

16. Li W, Godzik A. 2006. Cd-hit: a fast program for clustering and comparing large sets of protein or nucleotide sequences. Bioinformatics 22:1658-9.

17. Katoh K, Standley DM. 2014. MAFFT: iterative refinement and additional methods. Methods Mol Biol 1079:131-46.

18. Letunic I, Bork P. 2021. Interactive Tree Of Life (iTOL) v5: an online tool for phylogenetic tree display and annotation. Nucleic Acids Research 49:W293-W296.

19. Viigand K, Visnapuu T, Mardo K, Aasamets A, Alamäe T. 2016. Maltase protein of *Ogataea* (*Hansenula*) *polymorpha* is a counterpart to the resurrected ancestor protein ancMALS of yeast maltases and isomaltases. Yeast (Chichester, England) 33:415-432.
